# Exploring the Use of an Augmented Reality Device Learning Tool for Multidisciplinary Staff Training on Domestic Abuse and Sexual Violence: Postintervention Qualitative Evaluation

**DOI:** 10.2196/60075

**Published:** 2025-03-19

**Authors:** Dilroshini Karunaratne, Jessica Whittock, Amber Moore, Krishna Dasigan, Jasmine Chevolleau, Brent Bartholomew, Nikki Kelly, Charlotte E Cohen

**Affiliations:** 1 Department of Medical Education, Chelsea and Westminster Hospital NHS Foundation Trust, London, United Kingdom; 2 Department of Safeguarding Adults and Children, Chelsea and Westminster NHS Foundation Trust, Standing Together Against Domestic Abuse, London, United Kingdom; 3 Department of Acute Medicine and Emergency Services, Faculty of Medicine, Chelsea and Westminster Hospital NHS Foundation Trust, London, United Kingdom; 4 The SafeLives Practice Team, SafeLives, London, United Kingdom; 5 Department of Genitourinary Medicine and HIV Services, Faculty of Medicine, Chelsea and Westminster Hospital NHS Foundation Trust, London, United Kingdom

**Keywords:** augmented reality, virtual reality, medical technology, domestic abuse, sexual violence, medical education, training, domestic violence, violence, assault, victim, survivor-centered, staff, community stakeholders, social care, innovation

## Abstract

**Background:**

Legislative policies published by National Health Service, England and the UK Government focus on prioritizing the creation of a stronger system. These frameworks emphasize on the improvement of health care staff’s ability to identify and refer domestic abuse (DA) survivors as key areas for supporting workforce development. Health care staff are often the first professional contact of survivors of DA, and insufficient staff training is a key barrier to survivors being identified and directed to support. The Microsoft HoloLens2 is a mixed-reality headset that allows virtual objects (holograms) to be integrated into the real world. Mixed-reality headsets are being increasingly used within medical education and have the advantage of independent operation, reducing the staffing requirements for teaching. The HoloLens2 can be used to project HoloPatients (HPs), which resemble clinically unwell patients, into the classroom. Two of these HPs have been specifically designed to portray survivors of DA and sexual violence (SV).

**Objective:**

This study explored potential uses of the HP in DA and SV training as a potential survivor-centered educational initiative that could be used as an adjunct to existing training for health care professionals and community sector workers.

**Methods:**

Frontline staff and community stakeholders from the national health service, DA, and law enforcement sectors were invited on 3 separate occasions (n=14, 12, 22) to a HoloLens2 demonstration that displayed 9 HPs. The patient voice was to be outlined by personalized scripts, co-created alongside sector charities, ensuring survivor engagement and participation. Participants were given the opportunity to wear the headset and familiarize themselves with the technology during the sessions. A post-intervention evaluation research model was used to explore the feasibility and functionality of the HP as an educational tool.

**Results:**

Thematic analysis described the HP as a “realistic,” “adjustable” tool that “creates a safe learning environment.” Participants suggested it could be useful in “pre-exposure preparation” by “improving communication” and allowing different approaches to be trialed in a safe environment. The use of survivor scripts was described as a useful tool to “bring the survivor into the learning space” in a safe way. Participants identified the HP as a suitable tool for workers inside and outside health care, including social sectors such as law enforcement (32%).

**Conclusions:**

The HP acts as a low-risk, adaptable tool for trainees to develop skills in a safe environment. This study demonstrates that professionals perceived the HoloLens as an innovative means to amplify the lived experience voice. Further research will evaluate this additional impact on trainees’ confidence and responses to survivors disclosing DA and SV within different disciplines to drive improved outcomes.

## Introduction

In the UK, it is estimated that nearly 16.6% of adults aged 16 years and older have experienced sexual assault [1]. Furthermore, it is estimated that 2.1 million people aged 16 years and older experienced domestic abuse (DA) in the year ending March 2023 [2]. These statistics are comparable to the number of patients currently living with type 2 diabetes mellitus [3]. However, unlike diabetes and other medical conditions, there is little evidence discussing the pathogenesis behind DA and sexual assault, thereby making prevention programs, both difficult to design and sustainably fund. With one of the highest rates of repeat victimization than any other crime [4], DA affects 1 in 5 adults during their lifetime [5]. In addition, the detrimental effects of DA are wide-ranging, encompassing physical, mental, behavioral, and reproductive consequences [6]. Survivors of DA seek health care more frequently than individuals who have not experienced abuse [7], varying from presentations related to the physical consequences of abuse to psychological health problems, including depression, anxiety, and post-traumatic stress disorder [8]. Therefore, health care providers have a key role in identifying and supporting survivors. A systematic review of barriers and facilitators to disclosure of abuse to health care professionals (HCPs) found that perception of the HCP’s ability to respond to the disclosure was impactful. Any notion that the HCPs did not possess the skills or knowledge to appropriately respond deterred survivors from disclosing their experiences [8]. HCPs are often the first point of contact for survivors; hence, it is vital that they are equipped with the skills and tools to competently identify and support survivors [9].

According to the UK Government, all behaviors, inflicted on anyone perceived to be of a controlling, coercive, harmful, or sexual nature can be defined as DA or sexual violence (SV) [10]. Whilst the majority of survivors of DA are women [2], it is important that we do not neglect the experiences of those from the LGBTQ+ (lesbian, gay, bisexual, transgender, and queer) community, who are also more vulnerable to familial abuse and honor-based violence [11]. There are many barriers to LGBTQ+ individuals seeking support, including discriminative attitudes from the police leading to mistrust, as well as a lack of training for professionals from the criminal justice system and public sector on how to recognize and respond to DA in the LGBTQ+ community [12]. Although the data for England and Wales for the year ending March 2024 showed no statistically significant differences in the prevalence of domestic violence between ethnic groups [2], the literature shows that Black, Asian, and minority ethnic individuals experience multiple barriers in seeking help following abuse, including structural racism, cultural exclusion, and victim-blaming from both formal services and communities [13]. Furthermore, the 2024 statistics for England and Wales showed that a higher proportion of people with a disability experienced DA than those without [2]. These issues should not be considered as separate entities, but rather an intersectional approach must be undertaken, so we can understand how inequalities due to race, sex, disability, class, and sexual identity may compound each other, further preventing survivors from seeking help [11]. These statistics outline the need for services to adopt a survivor-centered approach. Strategic partnerships consulting survivors of all protected characteristics are needed to enable the development of pathways specifically designed to address their psychosocial needs. This is outlined in The Strategic Direction for Sexual Assault and Abuse Services [14], which is an initiative from National Health Service (NHS) England, with the goal of radically improving access to services and support for survivors of sexual abuse to help them heal and rebuild their lives. This requires collaboration between authorities, health services and strategic partners in the voluntary and community sectors as part of a multiagency care model [15].

Existing literature suggests a gap in the knowledge of HCPs on how to identify and respond to survivors of domestic violence and sexual assault, largely due to a lack of training [16,17]. A 2020 metasynthesis of 47 qualitative studies examined health practitioners’ readiness to address domestic violence and abuse. Support from the health system was the largest theme, with HCPs suggesting further support was needed through upskilling staff in how to address domestic violence, as well as making enquiries into domestic violence routine and allocating additional time for sensitive conversations [16]. HCPs spoke about the importance of training in how to identify and respond to DA in their own specific clinical setting [16]. Evaluations of existing domestic violence support training programs for HCPs have demonstrated significant improvements in knowledge and confidence in how to identify and respond to survivors [17] and hence, we set out to design a training program adjunct, using a mixed reality device.

The emerging technologies of virtual, augmented, and mixed reality devices create an expansive new realm for education. Virtual reality is an immersive experience where a virtual world replaces the physical world, whereas augmented reality uses technology to superimpose virtual data (visual information) onto the physical world (real-life objects) [18]. Mixed reality is an in-between medium which allows virtual objects to be integrated and anchored onto the physical world [18]. The use of mixed reality headsets, such as the Microsoft Hololens2, is being increasingly used within medical education. There are many advantages to using these devices, including the option of repeated practice of learning, skills, and communication, without adverse effects on the patient [19]. These devices also can be used independently, reducing the time burden of teaching sessions on clinical educators. So far, mixed reality devices have been primarily used for teaching anatomy (through understanding of 3D object layers and components), procedures, and clinical skills [19-21]. We wanted to explore the benefits of using them for DA and SV training. In order to effectively teach HCPs about domestic violence and sexual assault, it is important to use examples based on real-life experiences of survivors. To ask a survivor to facilitate a training session could be considered unethical, as it may be triggering to the survivor, with potential negative effects on their mental health and well-being; hence, the use of mixed reality devices creates an opportunity to recreate these learning opportunities without adverse effects on survivors.

The Domestic Abuse team at Chelsea & Westminster Hospital NHS Foundation Trust adopts a “whole health” approach by pioneering a longstanding partnership between health care and community stakeholders [22] widely accepted as a best response in acute health. Their model centers on 3 key features: a co-located Domestic Abuse Co-ordinator (DAC) responsible for training workers in DA; the co-location of Independent Domestic Violence Advisors (IDVAs) to provide holistic and specialist support to survivors that are identified; and a DA Lead. Data suggest that survivors ‘felt safe’ and were more likely to report to an on-site IDVA while in the hospital [23]. Early identification of potential survivors by frontline workers is essential and stresses the importance of high-quality in-house training on the identification, management and referral of survivors of DA and SV.

The aim of this study was to improve the quality of staff training on the identification and management of DA and SV survivors. In collaboration with the Undergraduate Education Team at Chelsea and Westminster Hospital NHS Foundation Trust, the DA Team proposed the use of the Microsoft HoloLens2 as a tool to channel the survivor voice. This evaluation explores both health care and community stakeholder views of the augmented reality headset as an additional learning tool to promote survivor-centered training in a safe setting. We hypothesized that the following experience would demonstrate the Microsoft HoloLens’ potential as a teaching adjunct when educating staff on the identification and management of DA and SV survivors.

## Methods

We conducted a formative evaluation of the Microsoft HoloLens2, specifically the GiGXR HoloPatient (HP) application, as a training tool within the DA and SV landscape.

### Intervention

**Figure 1.**
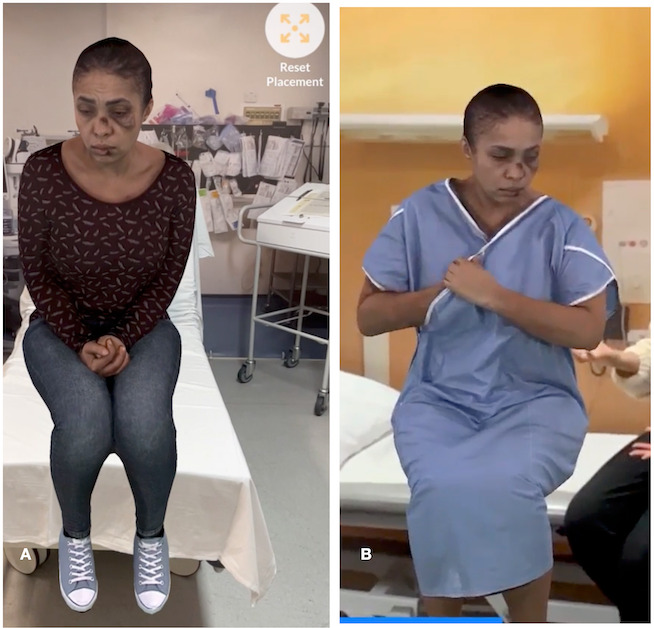
Lydia Johnson, a HoloPatient survivor of domestic abuse and sexual violence. Image A demonstrates Lydia on arrival to the emergency department. Image B displays her as a patient on a ward.

The Microsoft HoloLens2 is a mixed reality headset which has had a substantial impact in various industries since its release in 2019, including health care, education, and engineering [24]. Dissimilar to virtual reality, the headset is only part-immersive as it displays augmented content superimposed on the real surrounding environment. Organizations like GiGXR have created a series of augmented holograms, known as HPs, that resemble clinically unwell patients. The HP menu listed 17 holograms, each representing a clinical emergency, 2 of which were specifically designed to portray a survivor of DA (Lydia Johnson) and SV (Jenny Li). Each case consists of a series of video clips that depict the patient at different stages of their hospital journey (Figure 1). Aside from Jenny Li who vocalizes her experience via a prerecorded audio clip, all other holograms are nonverbal. As a result, the user has the flexibility to adapt the HP by overlaying a different patient script to suit their educational needs. This application, if used effectively, could expose students to unique training opportunities that are ethically challenging and difficult to recreate in a real clinical environment.

### Recruitment

**Figure 2.**
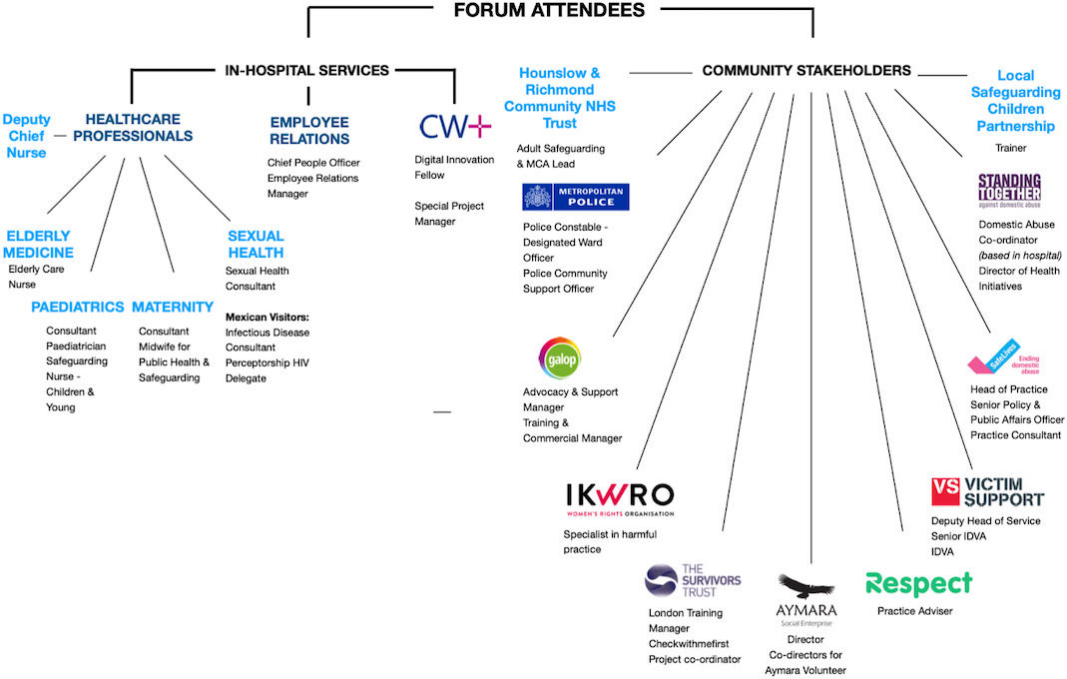
Spider diagram displaying all the organizations within North West London which attended the stakeholder forums.

The evaluation was conducted in the form of live demonstrations. These demonstrations were held within a lecture theater at Chelsea and Westminster Hospital. Frontline staff and community stakeholders from the NHS, DA, social care, and law enforcement sectors within the North West London area were invited to 3 HP events, held between February and July 2023 (see spider diagram displaying the range of attendees; Figure 2). Participants from these sectors were invited due to their increased likelihood of encountering survivors in a professional setting and their shared use of North West London Safeguarding guidelines. All participants were invited by email, which described the aim and method of the study in detail (see recruitment email in Multimedia Appendix 1). Participants were reminded that participation is voluntary and that all data were to be anonymized on publication. Written consent was obtained at the stakeholder meeting after participants were given the opportunity to read further information on the HoloLens and its purpose in this study (see consent form in Multimedia Appendix 2).

### The Demonstrations

**Figure 3.**
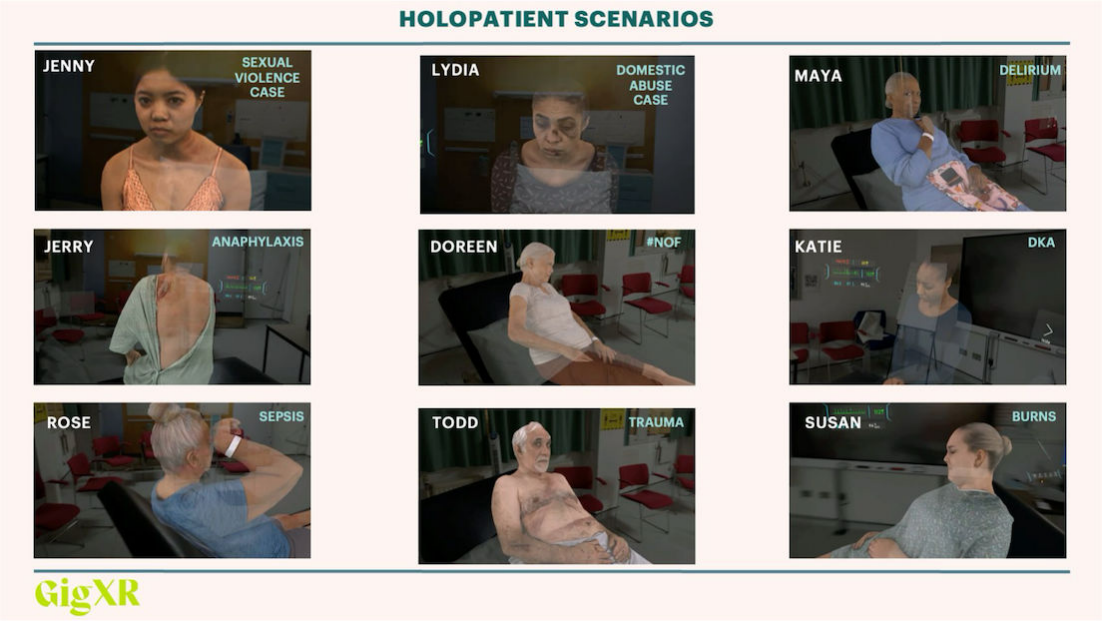
Nine HoloPatients selected to be displayed during the demonstrations. Their original clinical presentation demarcated in blue writing within each box.

Following a brief introduction into augmented reality, attendees were shown data that evidenced the HP as an immersive tool when trialed in an undergraduate setting [25]. The HoloLens was ultimately proposed as a teaching adjunct to be used whilst training frontline workers on the management of DA and SV cases (see full presentation in Multimedia Appendix 3). It was envisaged to be a visual aid that amplified the lived experience, to be used after their initial training with the DAC. The patient voice was to be outlined by personalized scripts, co-created alongside sector charities, ensuring survivor engagement and participation. In total, 9 HPs, different in gender, age, and ethnicity, were projected into the room (Figure 3). Participants were given the opportunity to wear the headset and familiarize themselves with the technology during the sessions.

### Data Collection and Analysis

A postintervention evaluation research model was used to explore the feasibility and functionality of the HP as an educational tool within DA and SV training. Furthermore, it evaluated the potential for a centralized multiagency augmented reality hub for professionals to access educational opportunities in the future. In order to achieve this, participants were prompted to answer 3 overarching questions: why should the HP be used, what should the HP be used for, and who should use the HP?

**Figure 4.**
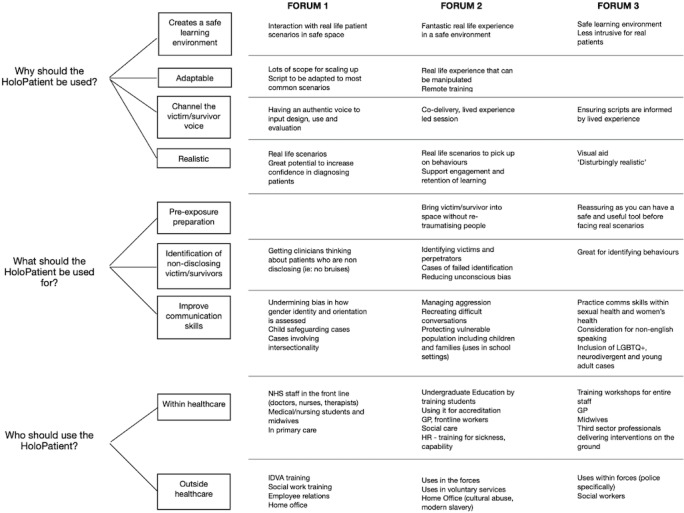
Coding tree across all 3 stakeholder forums.

Data were collected in the form of a standardized questionnaire to improve reliability (see in Multimedia Appendix 4). It consisted of Likert scaling [26] and open-ended questions to collect quantitative and qualitative data, respectively. Likert scales were used for participants to rate how strongly they agreed or disagreed with the following statements: “HoloLens is an effective tool used in DA and SV,” and “Technological limitations prevent me from using this tool.” Open-ended questions gave participants the opportunity to provide feedback on how they felt wearing the device, if they thought it would be useful in health care or other fields, and if they felt there was value in having a centralized multiagency HoloLens training hub. A basic analysis conducted on the Likert scale questions displayed statistical means and modes. The qualitative data were thematically analyzed using Braun and Clark’s 6-step method [27] (see data sets in Multimedia Appendices 5, 6, and 7). One researcher (medically qualified) read each survey repeatedly to gain familiarity. Initial interpretations were discussed with the project supervisor, and together they extracted evidence from the surveys to refine each theme. Finally, a list of themes was presented with illustrative examples on multiple occasions to the wider team (see coding tree in Figure 4). As a final checking step, draft findings were discussed with a few participants (n=3) prior to publication. To ensure that this study was conducted in accordance with qualitative reporting guidelines, a COREQ checklist was completed (Checklist 1) [28].

### Ethical Considerations

This evaluation was approved by the Research Innovation and Quality Improvement board of Chelsea and Westminster Hospital NHS Foundation Trust under reference number 6038. Written informed consent was obtained from all participants. All study data are anonymous, and HP scripts, although informed by DA and sexual assault survivors, did not contain any identifiable information. Involvement in this study was optional and voluntary, and there was no financial compensation for participants or researchers.

## Results

### Thematic Analysis

A total of 48 questionnaires were collected across all 3 demonstrations (n=14, 12, 22). Approximately 29% (n=14) of the sample were local HCPs. The remainder consisted of representatives from community charities, local councils, and law enforcement (see spider diagram displaying range of attendees in Figure 2). No participants refused to participate or dropped out.

Analysis of participants’ perspectives of the HP as a training tool in DA and SV sectors was divided into 3 overarching themes, based on our research questions: Why should the HP be used? What should the HP be used for? Who should use the HP?

A summary of this section is displayed in Table 1.

**Table 1.** Realistic table summarizing qualitative data following thematic analysis

Theme	Reason
Why should the HoloPatient be used?	
Realistic	“disturbingly realistic” “impactful” “increased their sense of reality” improving “retention of learning” “more realistic than role-play”
Creates a safe learning environment	brought “real-life learning experiences into a safe environment” trainees can repeatedly interact, make errors, stumble openly discuss case with peers without risk of re-traumatization
Adaptable	allows “real-life experiences to be manipulated” “scenarios adapted to each trainee’s requirement” “helpful for all professionals’ remote feature allows “scope to scale up”
Channel the survivor voice	Use of survivor script “essential” feature that could “bring the survivor into the learning space” to “incorporate language and cultural specific aspects” “inclusion of survivors when creating the stories” cover “common scenarios in different clinical settings and diverse cases”
What should the HoloPatient be used for?	
Pre-exposure preparation	“safe and useful tool for practice before facing real-life scenarios” “important to understand how to deal with the situation before interacting with survivors” law enforcement felt this could “support junior officers responding to their first call outs in a safer space.”
Identification of nondisclosing survivors	prepare trainees to identify survivors presenting without visible injury “masking patients” those “with a hidden agenda” “great for identifying behaviors or visible indicators”
Improving communication skills with and when	
Vulnerable patients	elderly mental illness neurodiversity women young people NHS staff or other staff
LGBTQ + survivors	“look at undermining bias in how people are assessed in regards to their their gender identity and sexual orientation” support exploration of “intimate partner violence” and “hate crime” have an “LGBTQ + lived experience voice” in the scenarios
Engaging with perpertrators	“look at undermining bias in how people are assessed in regards to their their gender identity and sexual orientation”
Scenario could be adapted to	where the survivor is “aggressive and protective of the perpetrator” “on how to approach a survivor and respond appropriately with a perpetrator present”
Recreating difficult conversations	“practicing mental capacity assessments” “decision-making, end-of-life care planning” “dealing with confrontation and bullying and harassment cases” demonstrate “an example of an incorrect way to deal with a situation” encourage “open discussion”
Who should use the HoloPatient?	
Within health care	
Undergraduate education	“medical and nursing students” training “first with the HoloPatient and then a certificate before the real practice”
Maternity	“the risk of domestic abuse during pregnancy” “trust building that happens between patient and midwife”
Other frontline staff	‘all health professionals who may come into contact with a survivor’ ‘no them and us’ targeting ‘third sector professionals who intend to deliver interventions on the ground’ could be used in IDVA^a^ training to ‘significantly reduce the time taken to train’
Outside of health care	
Social care sector	social workers paid carers other care home staff
Public sector	within law enforcement Home Office to recreate cases involving “minority ethnic survivors,” “honour-based abuse” and “modern slavery.’

a IDVA: Independent Domestic Violence Advisor

### Why Should the HoloPatient Be Used?

#### Realistic

Many attendees found the HP “disturbingly realistic” (Participant 1 (P1)) and were impressed with how “impactful” it was (P2, P3, and P4) (participant references taken from data sets—see Multimedia Appendices 5, 6, and 7). Attendees found that the HP “increased their sense of reality” (P5 and P6) hence improving engagement and “retention of learning” (P7). One participant found the HP “more realistic than role-play” (P2), as dynamics could be affected if a trainee is familiar with their actor or mentor.

#### Creates a Safe Learning Environment

Participants felt the HP brought “real-life learning experiences into a safe environment” (P8), where both trainees and survivors are better protected. In contrast to role-play, trainees can repeatedly interact, make errors, stumble, and openly discuss the HP’s case with peers without risk of re-traumatization.

#### Adaptable

Attendees acknowledged that the HP allows “real-life experiences to be manipulated” (P9) by using “scenarios adapted to each trainee’s requirement” (P1). As a result, a majority of participants saw it as a tool helpful for all professionals. HP demonstrations were live-streamed via Microsoft Teams to remote participants. One participant called this feature a “key benefit” (P10), which added “scope to scale up.”

#### Channel the Survivor Voice

The concept of the survivor script was presented to all participants at the HoloLens demonstrations. The scripts received positive feedback, with stakeholders seeing it as an “essential” feature that could “bring the survivor into the learning space” (P11). The idea was to overlay the HP scenario with a voice-over, either prerecorded or delivered by a survivor facilitator on the day. Participants particularly encouraged the use of the facilitator’s voice “to ensure that nuance isn’t lost” (P9). The potential to create scripts that cover “common scenarios in different clinical settings (eg, Emergency Department, sexual health)” (P12) and “diverse cases” with different protected characteristics were discussed. The idea to “incorporate language and cultural specific aspects” (P7) into the scripts was strongly considered. Attendees agreed with the “inclusion of survivors when creating the stories” to ensure that the scripts are “informed by a lived experience” (P3), with agreement that “specialist domestic abuse” (P2) input was necessary to maintain the use of trauma-informed language. The creation of “an authentic voice advisory group” (P13) for their overall evaluation was suggested.

Overall, all stakeholders strongly agreed (n=45) or somewhat agreed (n=3) that the Microsoft HoloLens 2 could be an effective tool to deliver training on the identification and management of DA and SV survivors.

### What Should the HoloPatient Be Used For?

#### Pre-Exposure Preparation

Stakeholders saw the HoloLens as a “safe and useful tool for practice before facing real-life scenarios” (P1). One participant described Jenny, the hologram of a SV survivor, as “incredibly uncomfortable to watch” (P14) explaining that it was “important to understand how to deal with the situation before interacting with survivors” (P14). Themes of realism and the HP’s ability to create a safe learning environment were commonly mentioned in conjunction with this theme. This is something that respondents cited clinicians often do not have access to, following any training. The HoloLens offers a training tool to embed the teaching from the DAC, before practicing in real life with patients. Law enforcement similarly felt this could support junior officers responding to their first call-outs in a safer space.

#### Identification of Nondisclosing Survivors

The HP’s potential to prepare trainees to identify survivors presenting without visible injury became a reoccurring theme across all demonstrations. One participant described the device as “great for identifying behaviors or visible indicators” (P2).

#### Improve Communication Skills

##### Communicating With Vulnerable patients

Once all the HPs were displayed, participants were prompted to suggest potential scenarios that could aid the identification of vulnerable survivors of DA or SV. Public Health England defines vulnerability as “in need of special care, support, or protection because of age, disability, risk of abuse, or neglect” [29]. Categorized into these subgroups, the suggested scenarios are listed below in ascending order of prevalence based on the questionnaire data: Elderly patients (n=2): “delirium,” “care-home scenarios”; women (n=4): “pregnant women,” “female genital mutilation”; patients with mental illness (n=7): “suicidal tendencies,” “eating disorders,” and “psychosis”; patients with visible and nonvisible disability (n=8): “scenarios that include adults with learning disability,” and “involving paid carers”; children and adolescents (n=16): “whole family scenarios,” “involving teachers,” “preparing families for court proceedings,” “forced marriage,” and “substance abuse”.

##### Communicating With LGBTQ+ Survivors

A participant stated that “generalist staff have not always got the skills to deal with LGBTQ+ survivors” (P15). In response, they suggested the potential use of the HP to “look at undermining bias in how people are assessed in regards to their gender identity and sexual orientation” (P15). They saw how this device could support exploration of “intimate partner violence” and “hate crime” by editing the scenarios to have an “LGBTQ+ lived experience voice” (P15). A few participants highlighted the absence of any transgender HPs (P1, P15, P16, and P17). When asked to suggest designs for new HPs, 22% (n=11) of participants said LGBTQ+ HPs.

##### Dealing With Perpetrators

In the first stakeholder meeting, a scenario was adapted to include a perpetrator by getting a facilitator to interact with the HP by standing in the background. They are visible to the HoloLens user and all participants watching the live stream. It was suggested that the scenario could be adapted to “challenge the stereotype of a passive survivor” (P9) where the survivor is “aggressive and protective of the perpetrator” (P9). Stakeholders saw its training potential within the police force (n=19), “specifically on how to approach a survivor and respond appropriately with a perpetrator present” (P3). However, concerns with safety were highlighted. One participant stated that “we need to be clear on the expectation around engaging with a perpetrator and how to do it safely if at all” (P9). This was seen as a key advantage of the HoloLens tool, as this could be explored in a safe space.

##### Recreating Difficult Conversations

Stakeholders listed “practicing mental capacity assessments, decision-making, end-of-life care planning, dealing with confrontation, and bullying and harassment cases” (P18) as challenging scenarios that could be practiced with the HoloLens. One participant suggested to display “an example of an incorrect way to deal with a situation” (P8) as a teaching tool to encourage “open discussion” (P18).

### Used in a Central Multiagency Training Hub

During the final demonstration, which took place in July 2023, attendees (n=22) were asked whether they supported the creation of a central multiagency training hub, potentially at Chelsea and Westminster NHS Foundation Trusts, which incorporated the use of augmented and virtual reality headsets. The intention of the hub is to provide community and health care services within the North West London region, shared access to the technology, and other training materials. This allows for a standardized training approach with greater opportunity for communication between teams. Overall creating more collaborative DA and SV multi-agency services in the future. A majority of participants either strongly (n=17) or somewhat agreed (n=2) in the creation of a central hub. It was stated that “sculpting multiagency responses into this (central hub) would be incredible and will change the health care landscape. The HoloLens can be used to continuously improve practice by dropping into an immersion center” (P4). One participant neither agreed nor disagreed with the hub, and 2 participants did not vote.

### Who Should Use the HoloPatient?

#### Within Health Care

##### Undergraduate Education

When asked where this technology could be most useful, approximately 14% (n=7) of participants suggested its use in undergraduate education. In particular, “medical and nursing students” (P5, P18, P19, P20, P21, and P22). One attendee suggested using the HoloLens for accreditation by training “first with the HP and then a certificate before the real practice” (P20).

##### Maternity

At least one participant per demonstration (total n=3) acknowledged its need in midwifery due to “the risk of DA during pregnancy and the trust building that happens between patient and midwife. Additional attendees (n=5) saw potential for the HP to be used in “IDVA training” (P4), indicating it could significantly reduce the time taken to train if this were used in routine practice. IDVAs are trained specialists that work with clients “from the point of crisis to assess, discuss, and develop safety plans”[30].

##### Other Frontline Staff

Approximately 52% (n=25) of attendees felt as though the technology was suitable for “all health professionals who may come into contact with a survivor” (P9, P23). “No them and us” (P24) was stated by one participant. Particular attention was given to primary care (n=6), emergency, and sexual health service staff. Other scoping included targeting “third sector professionals who intend to deliver interventions on the ground” (P7). One participant stated that training “could be in real-time with the holograms” (P5), providing clinicians with “authentic role-play” (P5) that was “less intrusive than real patients” (P5). This is a unique concept when compared to existing training programs, which mainly consist of digital modules at a beginner level [31].

### Outside of Health Care

#### Social Care Sector

At all 3 demonstrations, over one-third of attendees (n=16) named social workers, paid carers, and other care home staff as members of the public who would benefit from DA and SV training with the HoloLens.

#### Public Sector

Similarly, around 39% (n=19) of participants described the HP’s potential to train staff within law enforcement. Particularly those who “attend a domestic abuse emergency” (P12).

The use of the technology in the Home Office was discussed, especially to recreate cases involving “minority ethnic survivors” (P18), “honor-based abuse” (P2, P24, and P25), and “modern slavery” (P17 and P18).

## Discussion

### Study Findings and Comparison With Previous Works

The findings of this stakeholder evaluation indicate that despite minor technological limitations, all stakeholders saw the Microsoft HoloLens2 as a novel and effective tool to deliver training on DA and SV, within health, social care, law enforcement, and specialist settings. The data encourage the use of co-created survivor scripts alongside the HPs, to create a training program that amplifies their voice and lived experience. This enables the training to remain survivor-centered without needing a survivor present in the room as a facilitator. This minimizes the potential for adverse effects on the survivor.

A common theme across the groups was the ability to identify behaviors that signaled abuse in patients who did not have obvious physical signs of abuse such as bruising or other physical injury. The literature highlights how survivors often do not freely disclose abuse and often actively try to hide it [7], and hence being able to identify the more subtle signs is key to having good and comprehensive training on how to identify survivors. Positive feedback on the ease of use of the HoloLens device was received from all 3 groups, spanning health care workers, law enforcement, and the charity sector, demonstrating that this is a widely acceptable medium to deliver training.

Stakeholders suggested that the HPs were useful for pre-exposure preparation, which aligns with how we envisage it being used in the future. There was a strong theme of realism, with participants describing how the holograms had a profound emotional impact, which we hope will mirror some of the thoughts and feelings experienced when encountering survivors in real life. Enabling this first encounter to occur in a safe and simulated environment empowers participants to trial different approaches to managing the scenario without the risk of harm to a survivor so that they are better equipped to appropriately handle these situations in real life.

The existing literature clearly shows that one of the key barriers to recognizing and supporting survivors is a lack of training [12,16,17]. Ideally, this training needs to be embedded into the undergraduate curriculum of HCPs, so that doctors and nurses graduate with the skills to appropriately manage these encounters. We have demonstrated the potential of using the HoloLens2 in a professional setting and hope this could also be used as a tool for educating undergraduate health care students.

### Limitations

We discovered numerous limitations within our project, both related to the Hololens2 device itself and to the application. Wi-Fi connectivity poses a significant issue. One attendee stated that the HoloLens is a “useful tool when in a Wi-Fi range.” To open the HP application on the Microsoft HoloLens headset, the device must be connected to a high-speed wireless network. This is a key limitation, as internet connectivity is not reliable, especially in the community. To overcome this, the Undergraduate Education Team at Chelsea and Westminster Hospital has used the smartphone “Wi-Fi hotspot” feature to supply a mobile connection. If the HPs are to be rolled out as a training program more widely, then considerations will need to be made into the creation of a downloadable version that can be operated without an internet connection.

We also found the limited battery life of the headset was a barrier to producing longer or back-to-back training sessions. Microsoft estimate the HoloLens’ battery life to have 2‐3 hours of active use [32]. As both the Microsoft Teams and GiGXR HP applications were open during the demonstrations, it caused the device to malfunction at approximately 1.5 hours. This was highlighted as “an issue” by participants. One user felt that it hindered their ability to “maximize opportunity when using it in the training room.” Microsoft has stated that the devices are fully functional while charging [32]. Therefore, the Undergraduate Education Team inserted a portable battery pack into the USB-C port to prolong battery life during the demonstrations. Despite these technological limitations, 64% of participants felt as though it did not affect their opinion of the HoloLens. Overall statistics demonstrated that 14% stated otherwise. while 23% remained impartial. Another limitation of the device is that as it uses more power, it heats significantly. This poses potential safety risks and makes the device less comfortable to wear. To combat this, training sessions will need to be limited to shorter periods of time. We would suggest not using the device for more than an hour at a time.

A further limitation of the HP application was the lack of diversity of the characters. Most participants (roughly 57%, n=27) stated that they would have liked a wider variety of “visible clues” to represent intersectional characters [33]. For example, “different ethnicities, cultural headwear, visible disabilities, walking aids, transgender patients, pregnant women, and children.” Unfortunately, this would require the HP menu to be redesigned, at significant cost, which remains a limiting factor when considering scalability. However, augmented reality allows the hologram’s background to be adjusted to the user’s preference therefore walking aids and other visual accessories can be added, in an alternative manner or with a real-life person in the scenario, either as a friend, a family member or a perpetrator.

We must also consider limitations related to the study design. Data were collected as postintervention questionnaires. Although this provided insights into how the HPs could be used, the questionnaire was fairly brief, consisting of 13 questions. A focus group of a random sample of participants may have provided more detailed insights into the utility and acceptability of the Hololens2, and the HPs as a training tool for DA and SV. Furthermore, a larger sample size would allow for more reliable results.

### Conclusions

This technology has the potential to educate professionals on the use of trauma-informed language while challenging preconceived assumptions around protected characteristics in safe settings. Our evaluation has demonstrated that the Hololens2 is a widely acceptable tool for DA and SV training, that can be used as an adjunct to existing training programs. There is currently very little research on how virtual, mixed, and augmented reality devices can be used for training in DA and SV; hence, we hope the positive results from our evaluation will make way for further large-scale studies [34]. As the HoloLens2 can be operated independently, it gives scope for training sessions to be delivered with minimal staffing requirements.

Virtual reality companies like SimX have developed DA-specific virtual reality patient simulations. In the future we would want to evaluate the efficacy of these modalities as a teaching adjunct in the DA and SV space with the aim to create a core framework applicable to all future mixed and virtual reality technologies [35,36]. It could be of additional direct benefit to employee relation team training around sexual misconduct following the release of NHS England’s sexual safety organizational charter [37]. The goal will be to roll out augmented reality Holosurvivors as a standard teaching tool, in addition to DACs, supporting the best multiagency team public health response to DA and SV in the UK.

## Supplementary material

10.2196/60075 Multimedia Appendix 1 Sample recruitment email sent to stakeholders.

10.2196/60075 Multimedia Appendix 2 Sample participant information and consent form.

10.2196/60075 Multimedia Appendix 3 Powerpoint presentation delivered by Undergraduate Education Team during HoloLens demonstrations.

10.2196/60075 Multimedia Appendix 4 Sample questionnaire given to participants during the HoloLens demonstrations.

10.2196/60075 Multimedia Appendix 5 Questionnaire data from the first HoloLens demonstration (23/2/23).

10.2196/60075 Multimedia Appendix 6 Questionnaire data from the second HoloLens demonstration (26/4/23).

10.2196/60075 Multimedia Appendix 7 Questionnaire data from the third HoloLens demonstration (13/7/23).

10.2196/60075 Checklist 1 COREQ checklist

## Abbreviations

DA: domestic abuse
HCP: health care professional
HP: HoloPatient
IDVA: Independent Domestic Violence Advisor
NHS: National Health Service
SV: sexual violence
