# Supplementary material for: Exploring the Use of an Augmented Reality Device Learning Tool for Multidisciplinary Staff Training on Domestic Abuse and Sexual Violence: Postintervention Qualitative Evaluation
Source: JMIR Form Res. 2025 Mar 19;9:e60075. doi: 10.2196/60075 (PMC11941276; doi:10.2196/60075)
Supplement: Multimedia Appendix 1 [file formative-v9-e60075-s001.pdf]

RECRUITMENT EMAIL

Dear Colleague

We would like to invite you to an exciting training demonstration session on **Wednesday 22nd February 2-4pm at the Gleeson Lecture Theatre, LG Floor, Lift Bank C, Undergraduate Corridor (Imperial College sign by the entrance), Chelsea and Westminster Hospital, 369 Fulham Road, London SW10 9NH.**

Our Trust has been using a mixed reality headset (Microsoft HoloLens) to project holographic patients (HoloPatients) into our classrooms for medical students. By doing this we encourage students to interact with the hologram, assess the hologram, and create a management plan as if it were a real patient. This allows them to practice certain skills in a safe setting, for scenarios they may never have seen on the ward, particularly identification of abuse and communication with victim/survivors. Many students might not see such cases until their clinical placements or until after qualification.

Two of the holograms are patients who are victim/survivors of domestic abuse and sexual assault.

Our Domestic Abuse team is keen to partner with the Undergraduate Teaching Fellow, to evaluate the acceptability, and validity of this technology within our DA/SV training programme, both for undergraduate and postgraduate staff. We hope this could enable us to bring lived experience voices into the classroom using a recommendation framework, to improve the training experience, and responses to victim/survivors. Other HoloPatients designated for different scenarios, may also be useful and we will discuss this during the session.

Below is a Youtube link the academic team created, which demonstrates the HoloPatient functions.

https://gbr01.safelinks.protection.outlook.com/?url=https://www.youtube.com/watch?v=_QLqDKwi64c&data=05|01|dilroshini.karunaratne@nhs.net|266ded96295e4142ed6708db0f6691b4|37c354b285b047f5b22207b48d774ee3|0|0|638120705429144845|Unknown|TWFpbGZsb3d8eyJWIjoiMC4wLjAwMDAiLCJQIjoiV2luMzIiLCJBTiI6Ik1haWwiLCJXVCI6Mn0=|3000|||&sdata=q4SLZHy7Q/3Sx97kgolCKjU4oO+f/0qvjj/s1Vuw4dc=&reserved=0

We look forward to seeing you there.

Best wishes
Charlotte, Jess & Dilly

Dr Charlotte Cohen FRCP

Consultant Genito-urinary Medicine & HIV

Trust Domestic Abuse Lead

This is a Multimedia Appendix to a full manuscript published in the JMIR Formative Research. For full copyright and citation information see http://dx.doi.org/10.2196/jmir.60075.
