# Supplementary material for: Exploring the Use of an Augmented Reality Device Learning Tool for Multidisciplinary Staff Training on Domestic Abuse and Sexual Violence: Postintervention Qualitative Evaluation
Source: JMIR Form Res. 2025 Mar 19;9:e60075. doi: 10.2196/60075 (PMC11941276; doi:10.2196/60075)
Supplement: Multimedia Appendix 2 [file formative-v9-e60075-s002.pdf]

Information for participants in HoloPatient Domestic Abuse and Sexual Violence Training Project:

Date:

The HoloLens2 is a headset device which allows users to wear a camera on their head to stream to Microsoft Teams interface, approved for healthcare use and communication.

The HoloLens2 device has many projected uses, one of which involves medical/surgical education, and using it to teach procedures to undergraduates and postgraduates.

The Hololens2 device will be used in several teaching projects as described below at Chelsea and Westminster NHS Foundation Trust. The projects will involve collecting data from participants that may be analysed and published. Media taken of the projects ie: photos and videos, may be published online. If you do not wish to be included in the media please let us know.

Example of a HoloLens 2 device. The main streaming camera is shown here (red arrow)

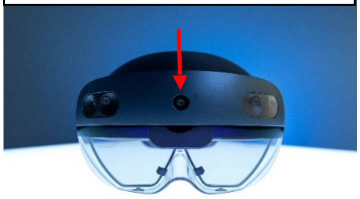

GIGXR HoloPatient Demonstration

The Hololens will be worn by the Education Fellows who will use the HoloPatient application to project holographic patients into the demonstration room. These holograms will represent victims/survivors of domestic abuse and sexual violence. You will be encouraged to assess and manage the hologram as if you would a real patient. The purpose of this application is to encourage participants to practice certain skills in a safe setting. The HoloPatient application does not contain confidential patient information therefore maintaining data governance.

Please be aware that this demonstration may display multimedia or discuss topics related to domestic abuse and sexual violence that could be distressing.

PARTICIPANT CONSENT FORM:

If you are happy to proceed with participating in the project involving the HoloLens2 please sign below:

'I have read this information sheet fully, have had the opportunity to ask questions and am willing to participate in projects involving the Hololens2 device.'

Name:

Signature:

Date:

'I have read this information sheet fully, have had the opportunity to ask questions and am happy to have media of myself participating in the Hololens2 projects to be published online.'

Name:

Date:

Signature:

This is a Multimedia Appendix to a full manuscript published in the JMIR Formative Research. For full copyright and citation information see http://dx.doi.org/10.2196/jmir.60075.
