# Supplementary material for: Exploring the Use of an Augmented Reality Device Learning Tool for Multidisciplinary Staff Training on Domestic Abuse and Sexual Violence: Postintervention Qualitative Evaluation
Source: JMIR Form Res. 2025 Mar 19;9:e60075. doi: 10.2196/60075 (PMC11941276; doi:10.2196/60075)
Supplement: Multimedia Appendix 3 [file formative-v9-e60075-s003.pdf]

USING THE HOLOLENS

TO CHANNEL THE SURVIVOR VOICE IN DOMESTIC ABUSE & SEXUAL VIOLENCE TRAINING

This is a Multimedia Appendix to a full manuscript published in the JMIR Formative Research. For full copyright and citation information see http://dx.doi.org/10.2196/jmir.60075.

Dr Charlotte Cohen
Dr Brent Bartholomew &
Undergraduate Education Department

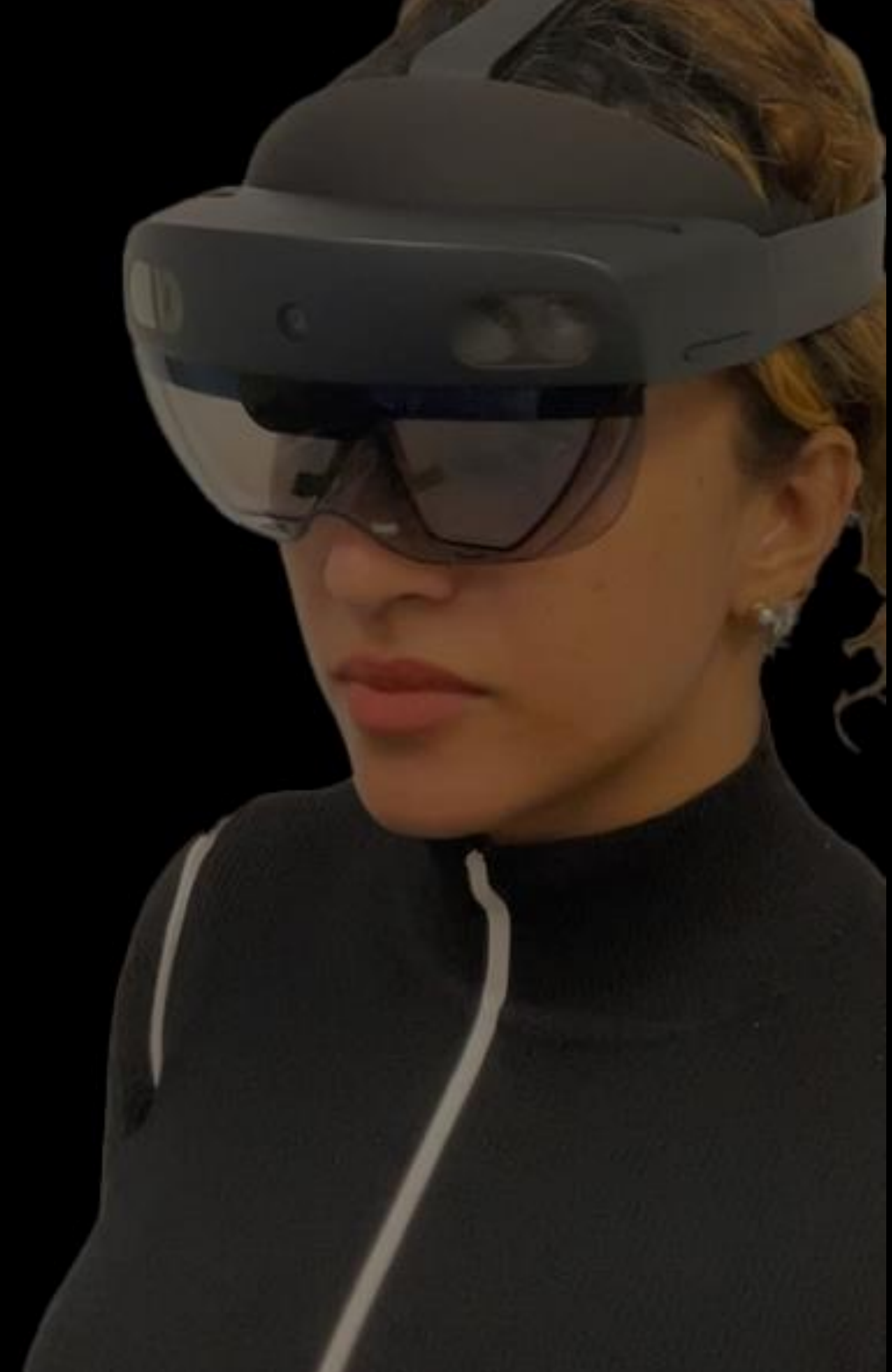

OVERVIEW

- Welcome and introductions
- Domestic Abuse landscape
- Key themes emerging from mapping
- Model of response at Chelsea & Westminster
- Multi-agency research proposal and next steps
- What is the HoloLens?
- Feedback from the stakeholder consultations so far
- Videos
- Refreshments and networking
- Playtime

Why are health initiatives so important?

- NHS Front Door
- Cost burden
- Domestic Homicide Reviews
- Hidden victims
- Mental health
- Hospital stays
- Key institution
- Legislative duties

COMPLEX LANDSCAPE

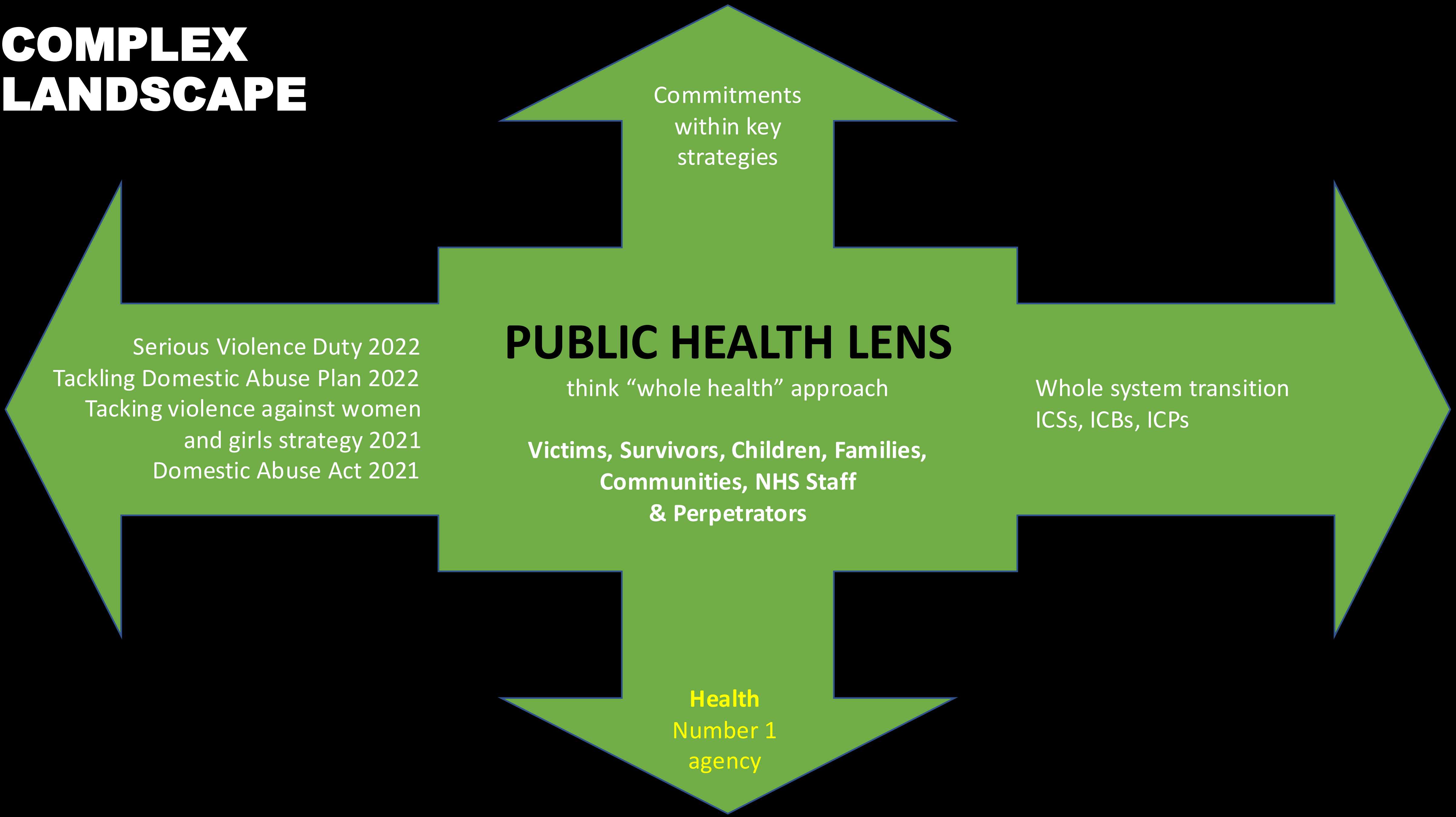

EMERGENT THEMES IN LONDON

- DA training not mandatory
- Perpetrator interventions
- Harmful practices
- Lack of consistent data recording, sharing and monitoring

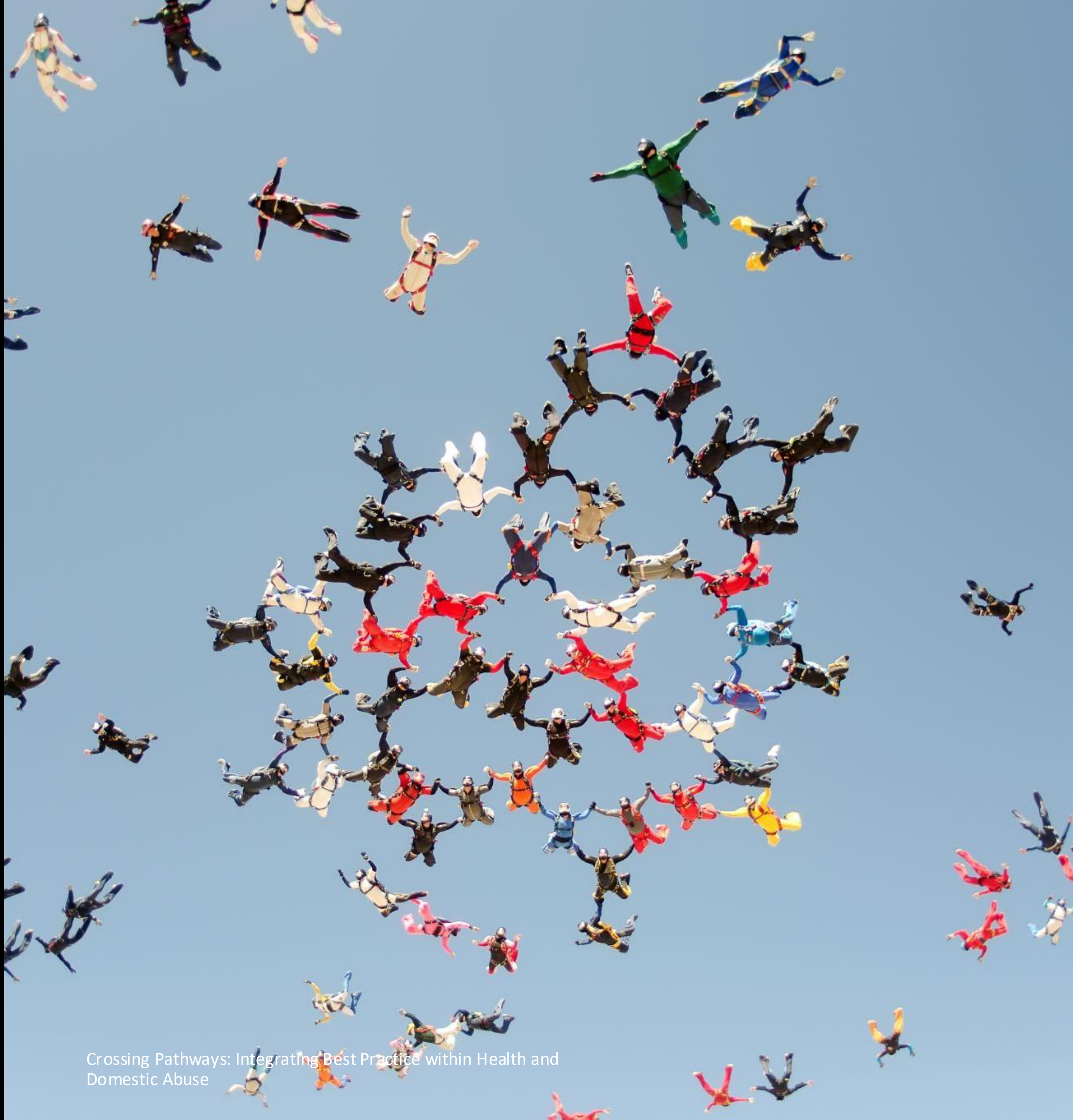

This is the image that needs changing colours to fit in and transposing

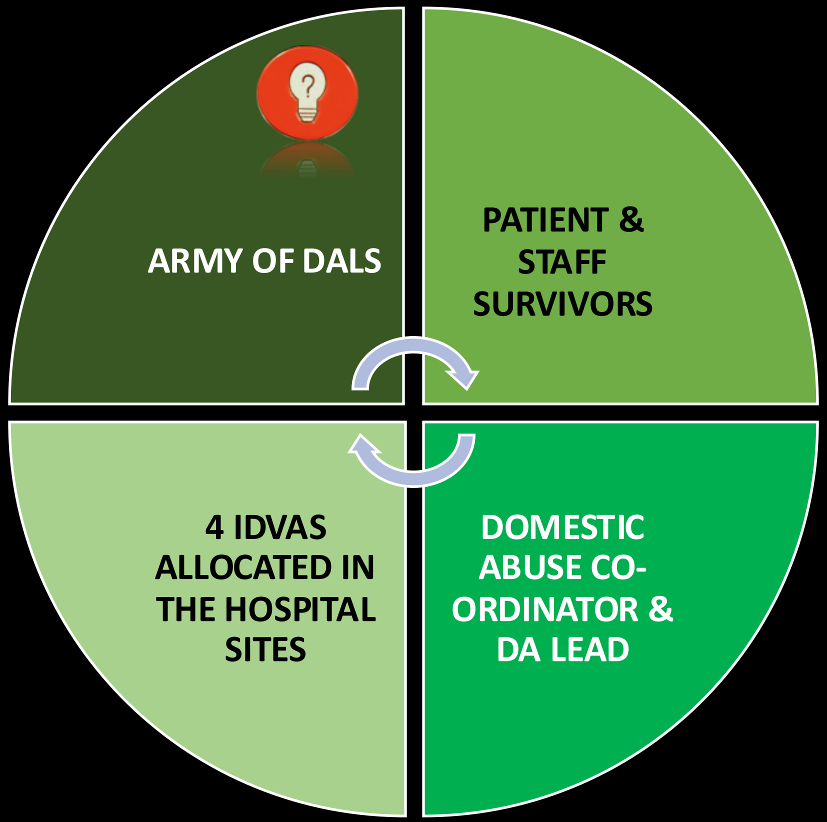

Trust Response Model

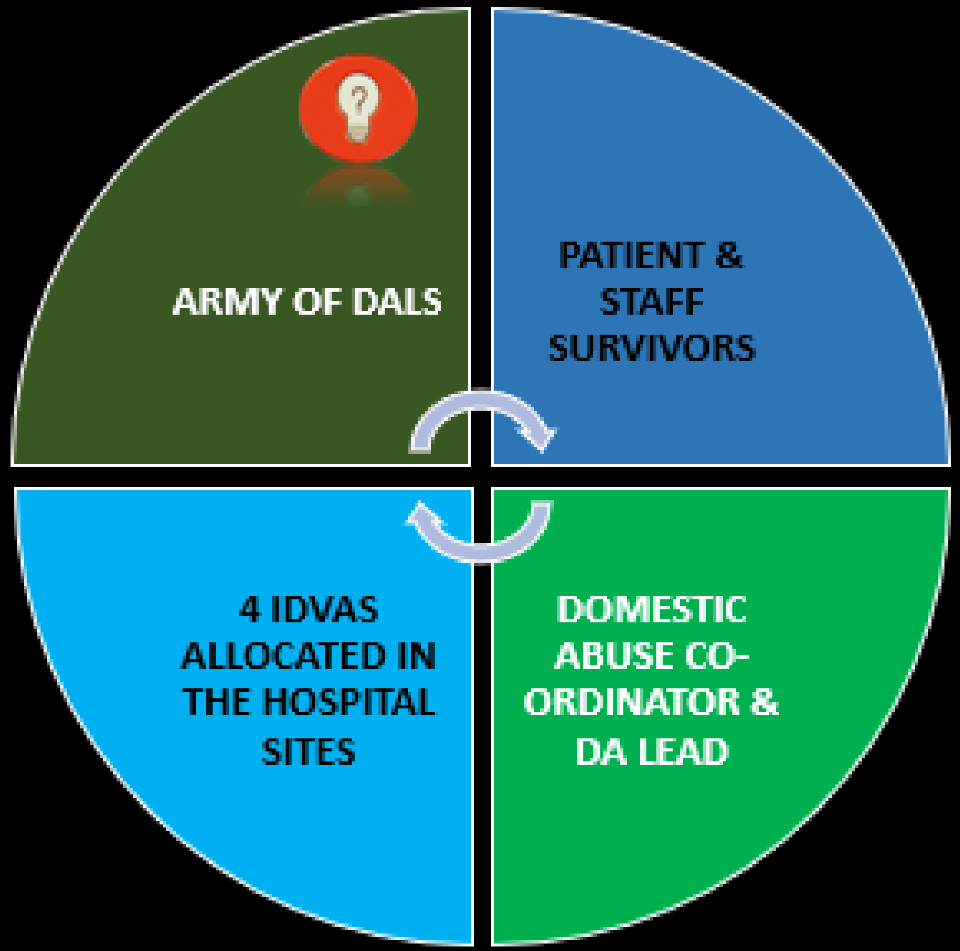

Missing areas

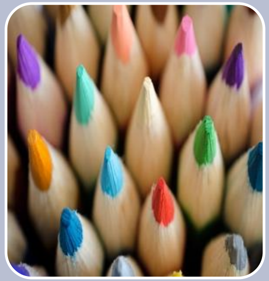 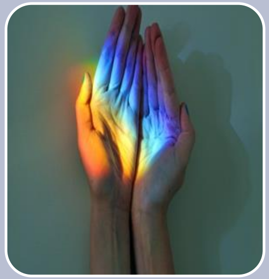 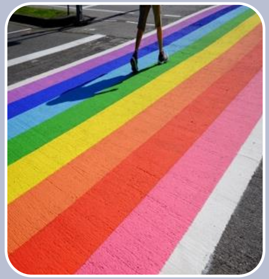 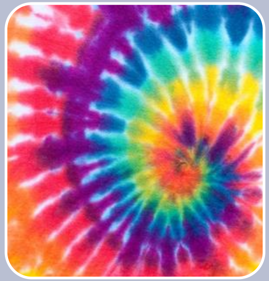 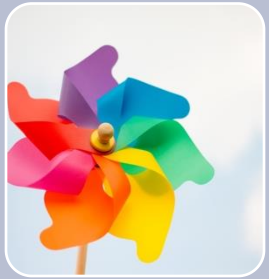

- ROBUST STAFF DA POLICY
- SURVIVOR ENGAGEMENT FORUM/ WORK
- PERPETRATOR IDENTIFICATION, ENGAGEMENT & REFERRAL PATHWAYS
- RELIABLE DOCUMENTATION POST-CERNER
- FURTHER EXPLORATION OF A MARAC LIAISON OFFICER ROLE

Training model

LEVEL OF TRAINING
**Level 1**<br>online bitesize training <1 hour
**Level 2**<br>F2F/online 1.5 hour basic awareness<br>tailored training for teams and departments including 1:1 for teams
**Level 3**<br>F2F/online whole day domestic abuse link (DAL) training
**Level 4**<br>Quarterly 2 hour supervision F2F/online<br>Continued professional development with additional specialist training<br>Looking at collaborations with CNWL to combine sessions with their DAC and DALs
**Level 4 Advanced**<br>Train the trainer courses for DALs<br>Whole day with ongoing support

This is the image that needs changing and transposing

Level of training
**Level 1**<br>online bitesize training <1 hour
**Level 2**<br>F2F/online 1.5 hour basic awareness<br>tailored training for teams and departments including 1:1 for teams
**Level 3**<br>F2F/online whole day domestic abuse link (DAL) training
**Level 4**<br>Quarterly 2 hour supervision F2F/online<br>Continued professional development with additional specialist training<br>Looking at collaborations with CNWL to combine sessions with their DAC and DALs
**Level 4 Advanced**<br>Train the trainer courses for DALs<br>Whole day with ongoing support

SURVIVOR-CENTRED TRAINING APPROACH

ACTION

- People who disclose that they are experiencing or perpetrating domestic violence or abuse **are offered referral to specialist services and an IDVA.**
- People experiencing domestic abuse and sexual violence receive a **consistent response from trained staff.**

ASSESS

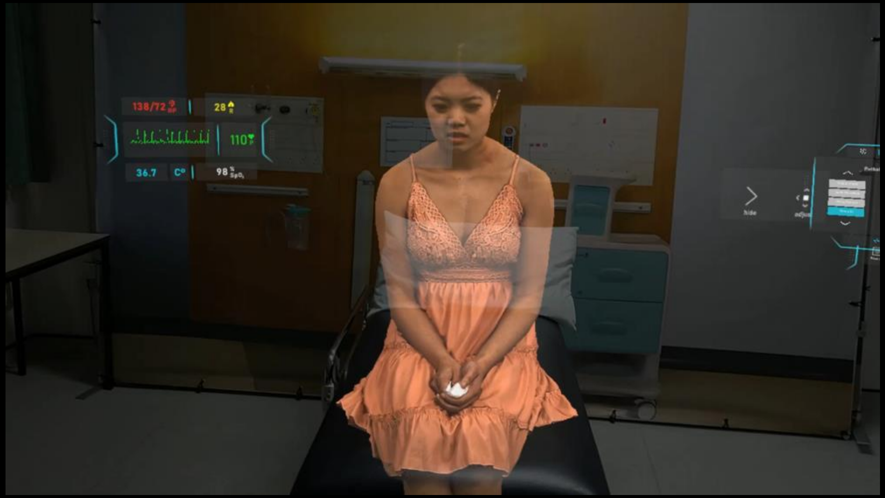

Bringing lived experience voices into the classroom

ASK

- People presenting to frontline staff with indicators of domestic abuse or sexual violence **are asked** about their experiences in a safe space

VALIDATE

- **Validation** of any disclosure is important, so the survivor knows this is not their fault and they are not alone.

Domestic violence and abuse
Quality standard [QS116] Published: 29 February 2016

NICE National Institute for Health and Care Excellence

HoloPatient Scenarios

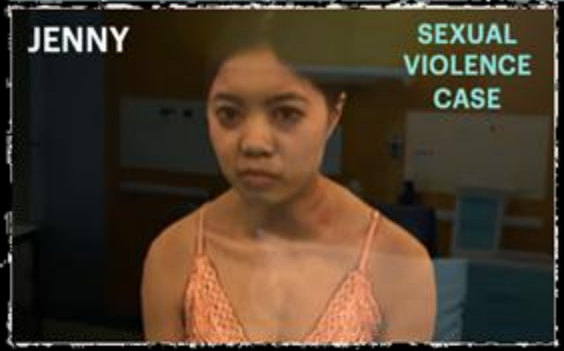

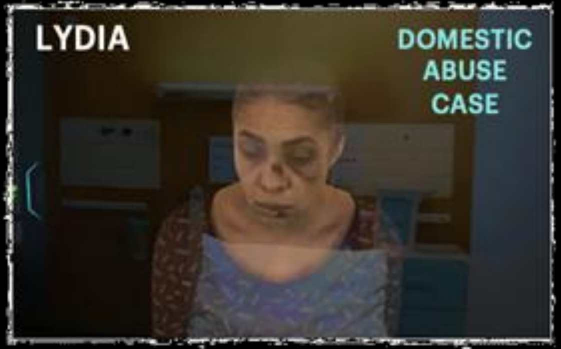

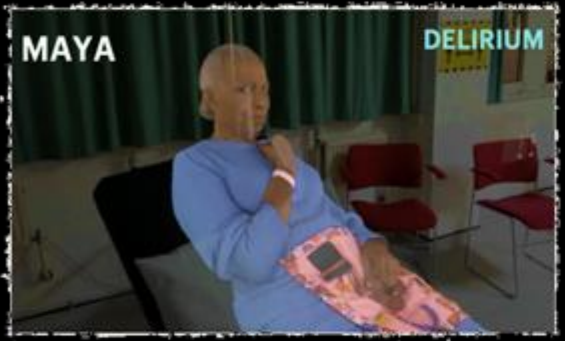

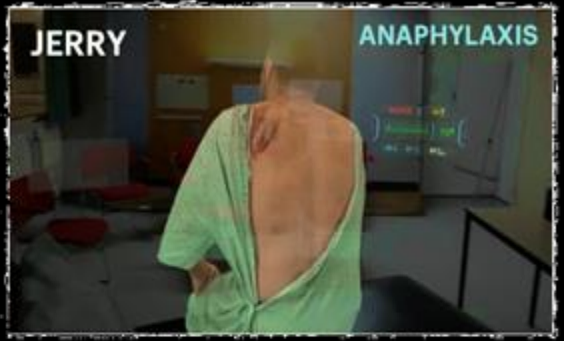

Customised scenarios

Burn
Anaphylaxis
Trauma
Domestic Abuse
Diabetes
COPD

Heart Failure
Stroke
Asthma
COVID-19
Dementia
Sexual Violence

Parkinson's
Septic Shock
Hip Fracture
Pre-eclampsia
Myocardial Infarction

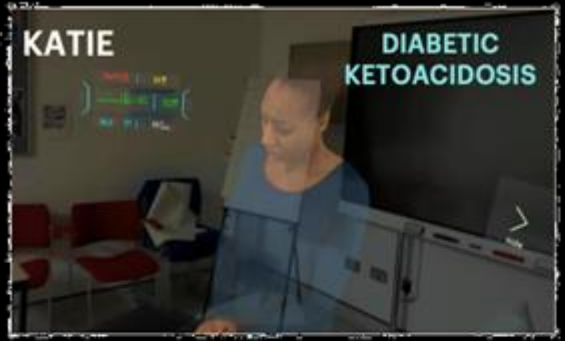

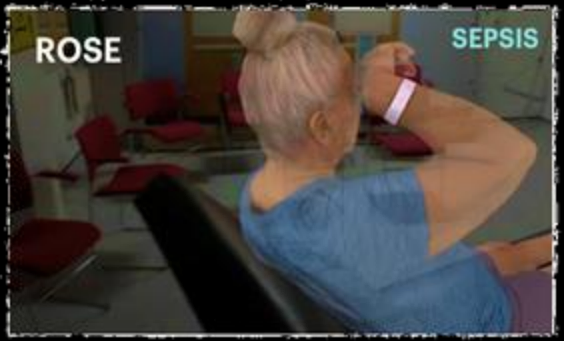

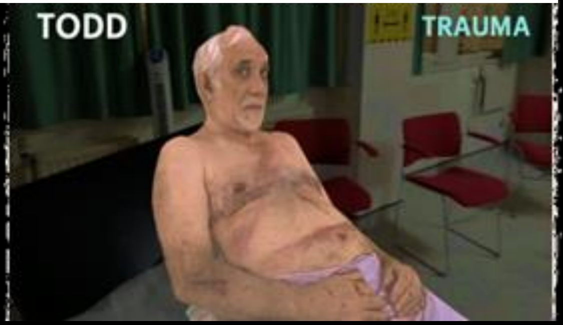

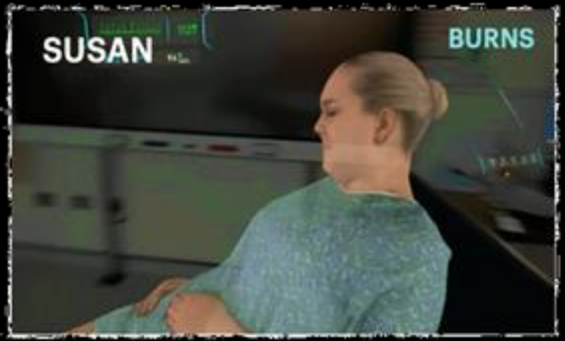

GigXR

NHS
Chelsea and Westminster Hospital
NHS Foundation Trust

Challenges

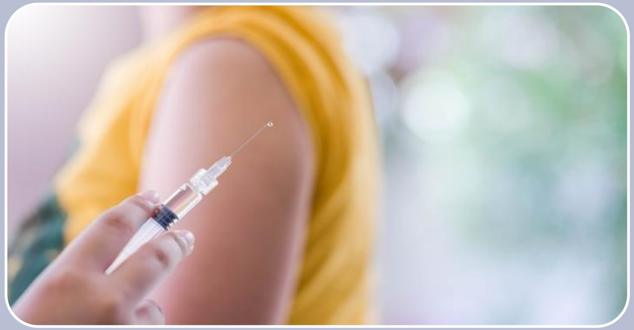

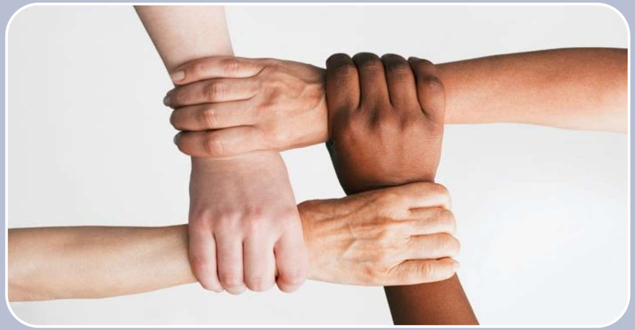

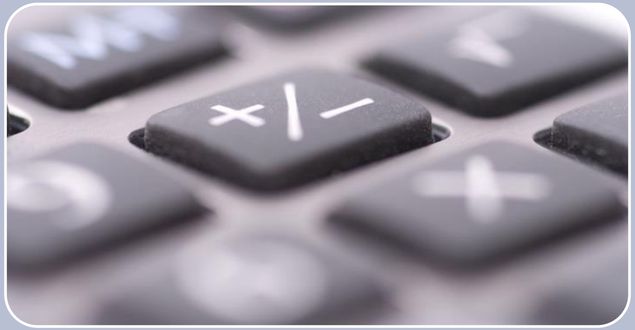

On-going post pandemic backlogs

Bed pressures

Competing health priorities

IDVA capacity

DA team space

Training capacity with staff turnover

Engaging with all staff

New IT systems

Working at system level in the ICS

Leading the way: next steps....

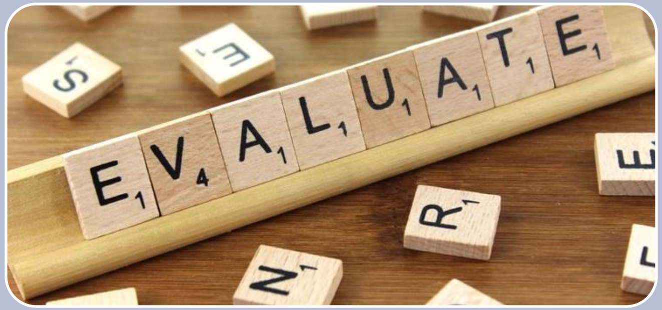

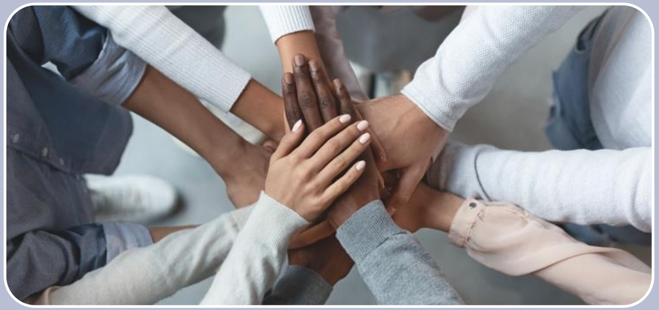

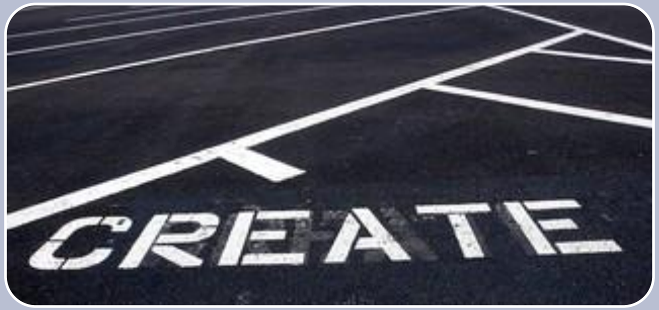

EVALUATE

organisational response to create **framework of recommendations around survivor voices**

COLLABORATE

with key stakeholders to **validate** the additional impact of HoloLens for **training and assessment** around DA and SV

CO-CREATE

scripts and tools to **amplify the lived experience voice** and improve multi-agency responses

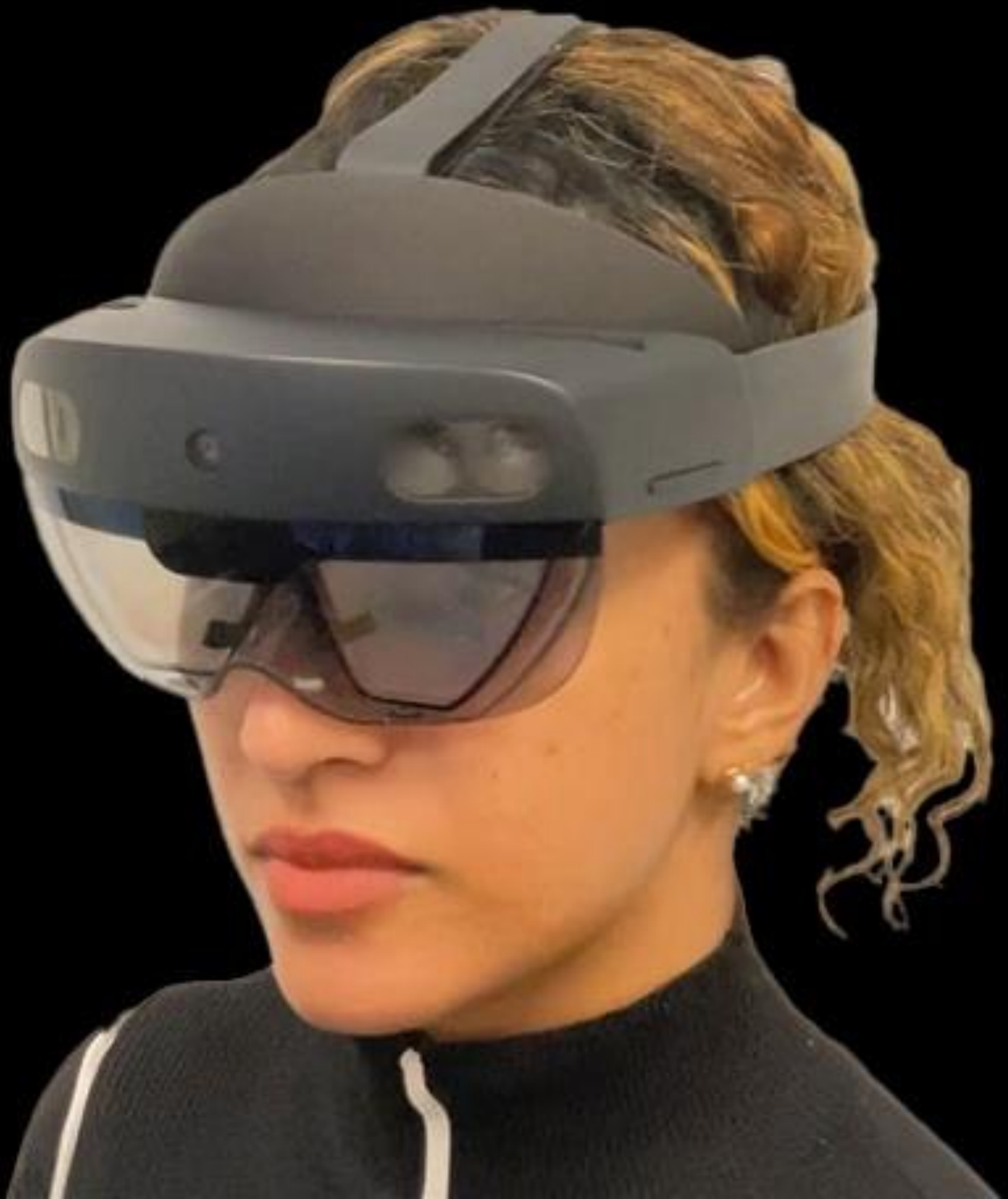

What is the Microsoft HoloLens2?

Undergraduate Education Team
Chelsea & Westminster NHS Foundation Trust

REMOTE ASSIST

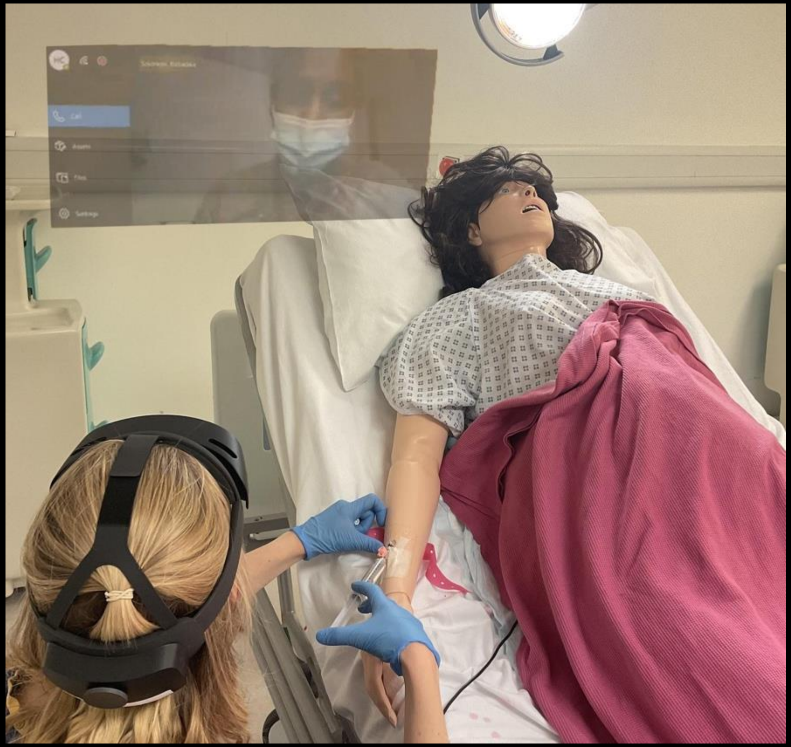

Enhancing
Care

DYNAMIC 365 GUIDES

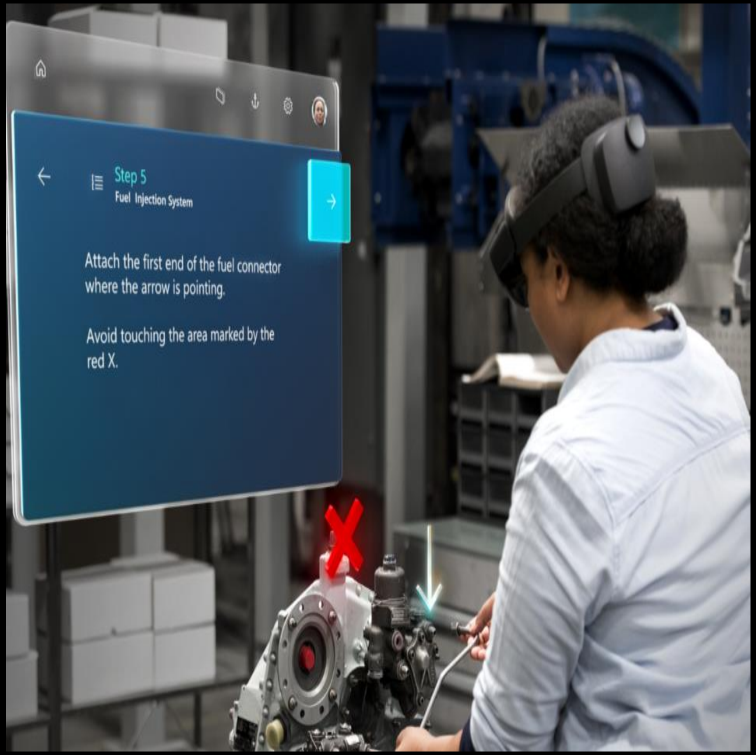

Empowering
Staff

GIGXR HOLOPATIENT

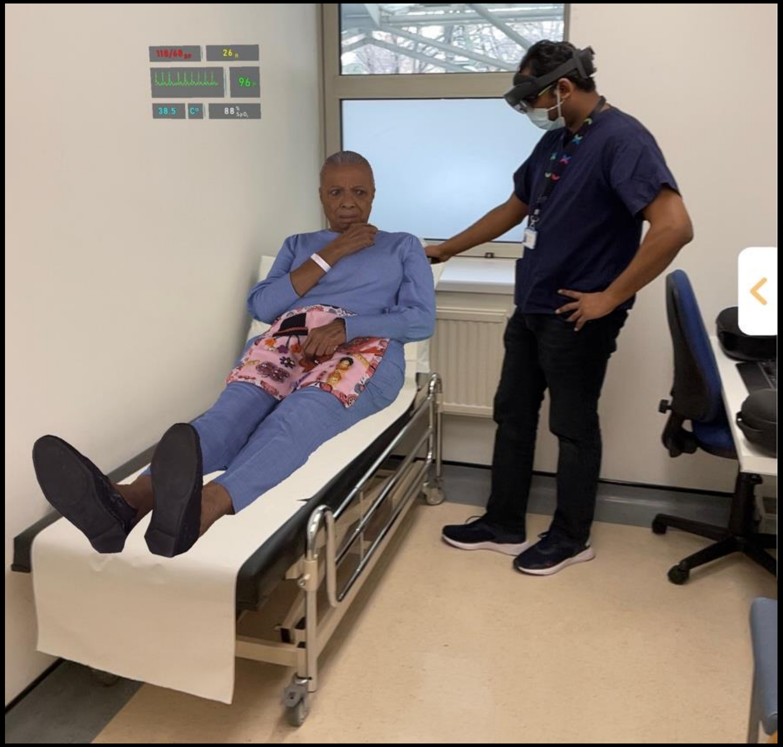

Breaking
Barriers

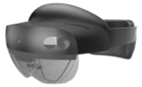

Enhancing Care, Empowering Staff, Breaking Barriers

NHS
Chelsea and Westminster Hospital
NHS Foundation Trust

HoloLens Projects

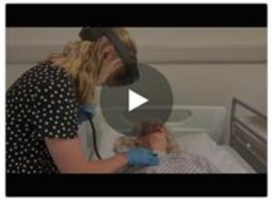

Incorporation into weekly Friday simulation: particularly the asthma/sexual assault Holopatient programmes

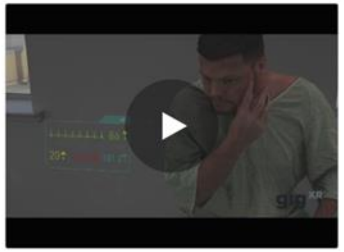

Procedures/examination skills

Paediatrics

Remote exposure to complex obstetric procedures

O&G

Using HoloPatient capabilities to facilitate teaching for midwives and senior O&G doctors - particularly the pre-eclampsia, domestic violence, asthma and diabetes HoloPatient programmes

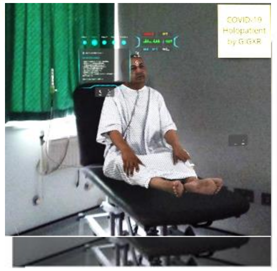

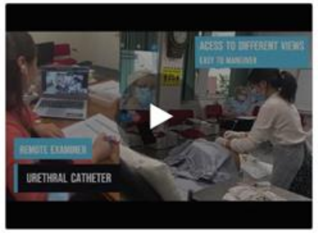

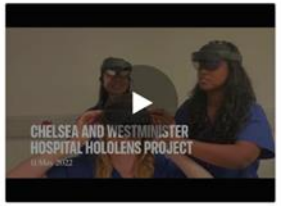

Remote[illegible]

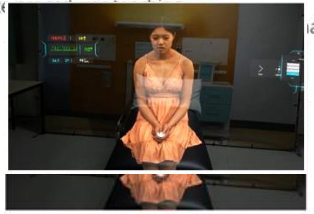

[illegible]atology

Holographic patients to facilitate domestic abuse training

Domestic Abuse Team

OSCE Examiners

Exploring the impact of the Hololens 2 365 function by the first examiner, to live stream final year medical students undertaking a formative clinical skills assessment to a second examiner

Exploring the impact of the Hololens 2 365 function by the examiner, to mark a holographic projection of the OSCE mark scheme

ITU/Anaesthetics

Remote expert consultation (especially in regional anaesthesia)

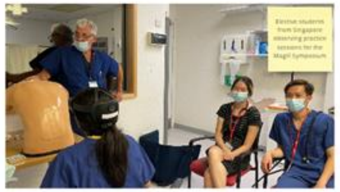

Holographic patient consultation (eg pre-eclampsia/eclampsia teaching)

Clinical skills and simulation practice as a means to bridge observed simulation practice and performing procedures on real patients

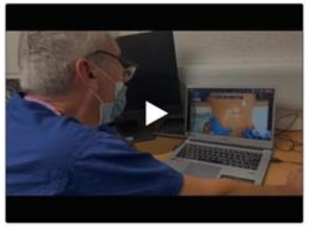

Cardiology

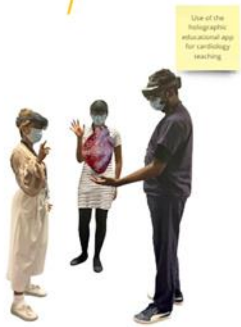

Remote echocardiogram teaching

A to E via HoloLens in final year medical student tutorial

Live stream bedside examinations to final year medical students to augment classroom teaching on common on-call scenarios

MRCP Practice

Remote access for trainees to live streamed MRCP practice sessions with real patients

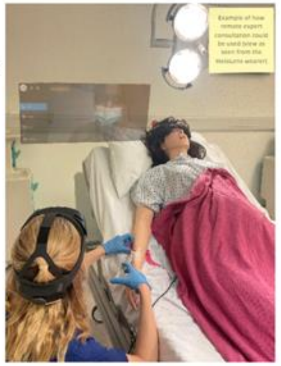

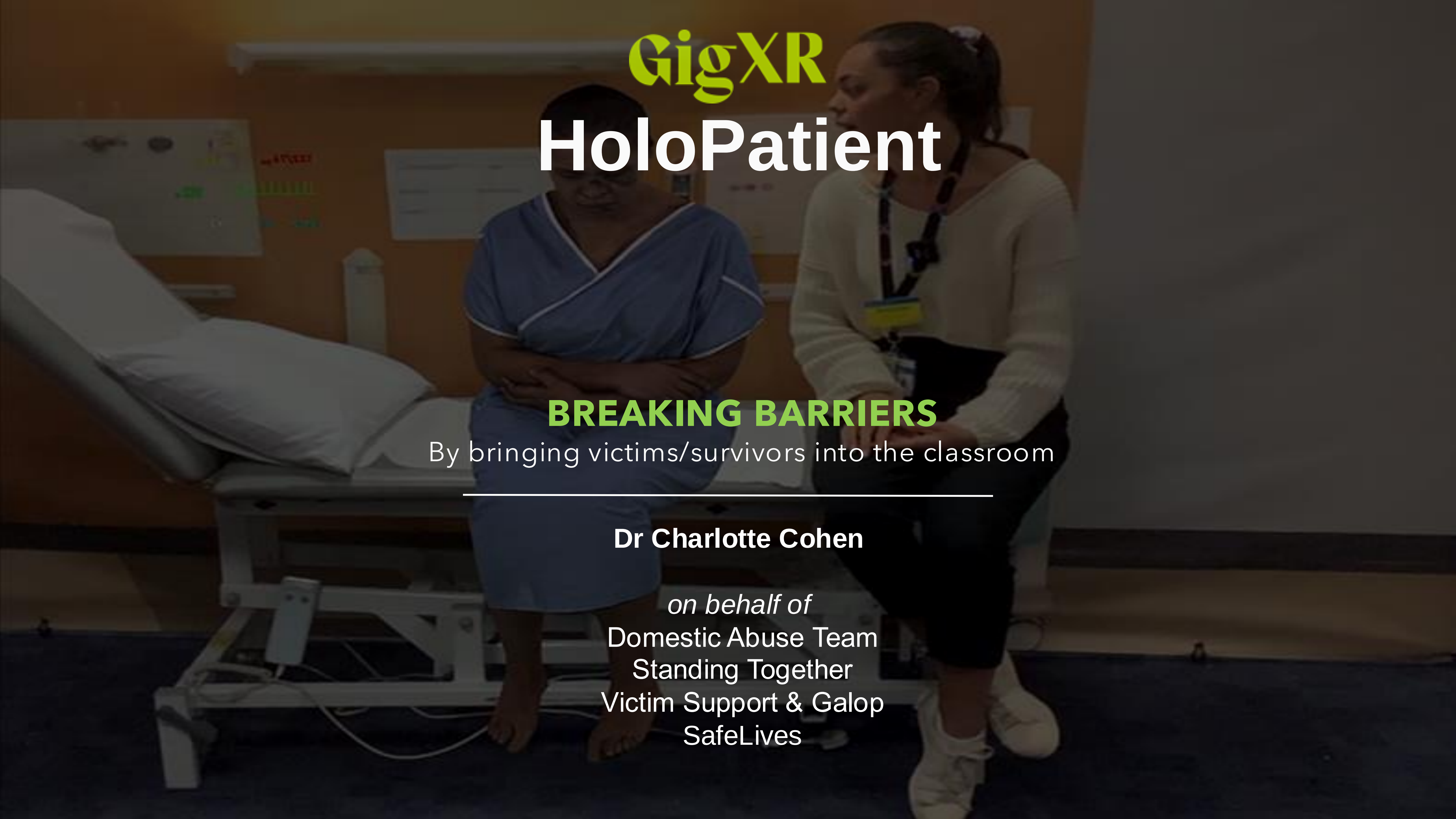

GigXR

HoloPatient

BREAKING BARRIERS

By bringing victims/survivors into the classroom

Dr Charlotte Cohen

on behalf of

Domestic Abuse Team

Standing Together

Victim Support & Galop

SafeLives

HoloPatient Demonstration

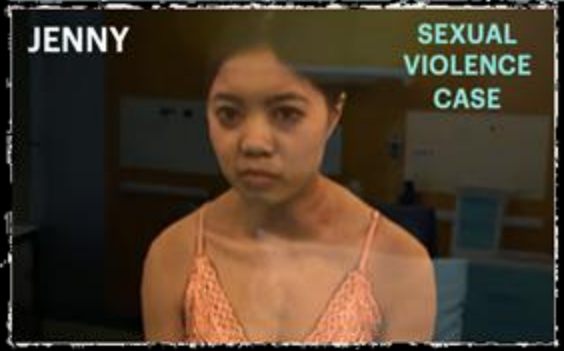

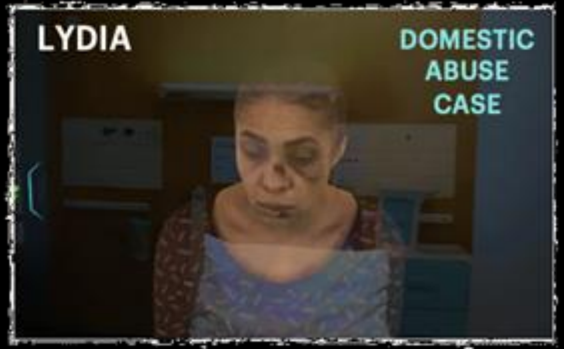

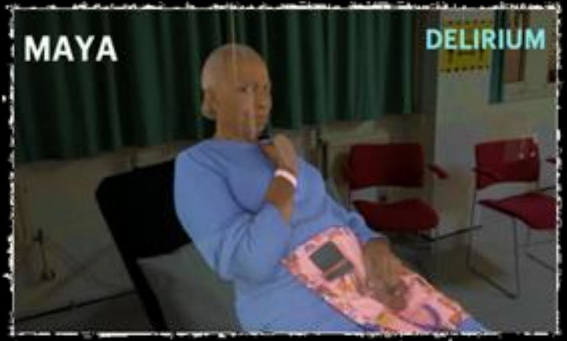

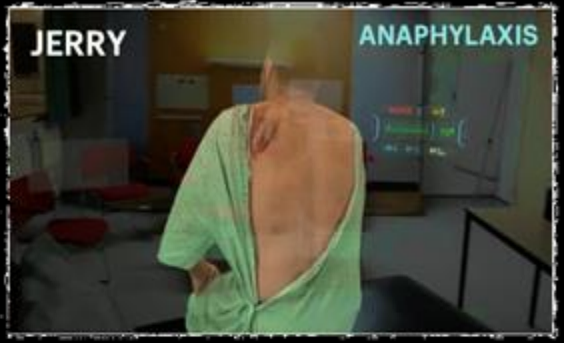

Customised scenarios

Burn	Heart Failure	Parkinson’s
Anaphylaxis	Stroke	Septic Shock
Trauma	Asthma	Hip Fracture
Domestic Abuse	COVID-19	Pre-eclampsia
Diabetes	Dementia	Myocardial Infarction
COPD	Sexual Violence	

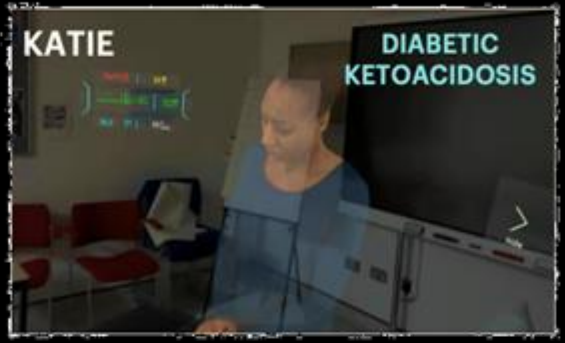

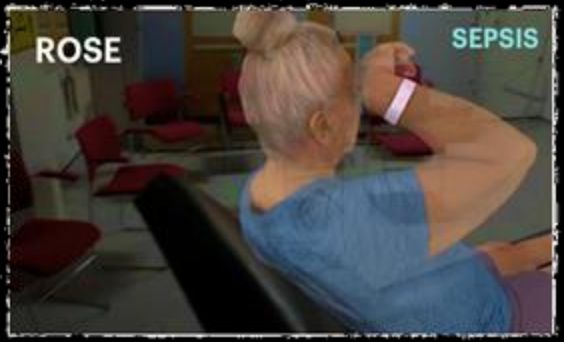

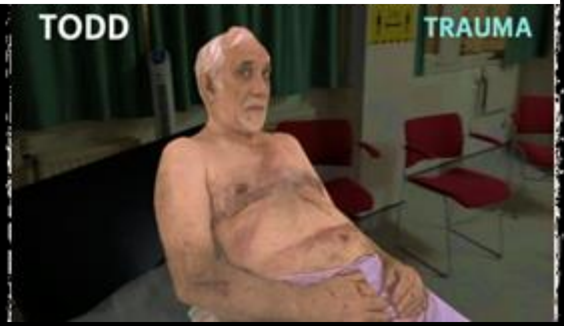

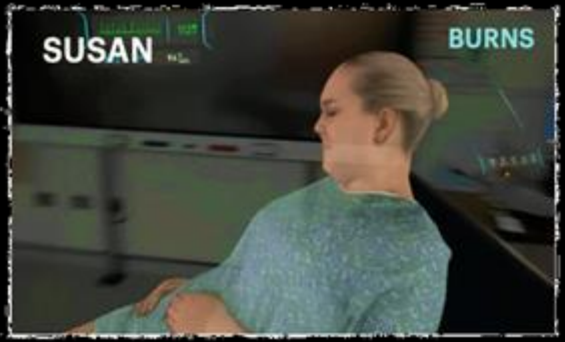

GigXR

NHS
Chelsea and Westminster Hospital
NHS Foundation Trust

LIMITATIONS

HOLOLENS

LIMITATIONS	SOLUTIONS
Frequent eye calibration	Can be temporarily disabled
Battery life (approx. 1 hour)	Can use external battery pack.<br>Could record videos instead.
Overheating	

HOLOPATIENT

LIMITATIONS	SOLUTIONS
Non-adjustable gender, ethnicity, age, speech, clinical signs	Customisable observations<br>**Commercial partnership (GIGXR/Undergrad Dpt) – develop new scenarios with an upfront fee**
Visual modality only: non-tactile, one-way conversation	Trainer can overlay script. Can stop/start/replay scenario.

Stakeholder engagement

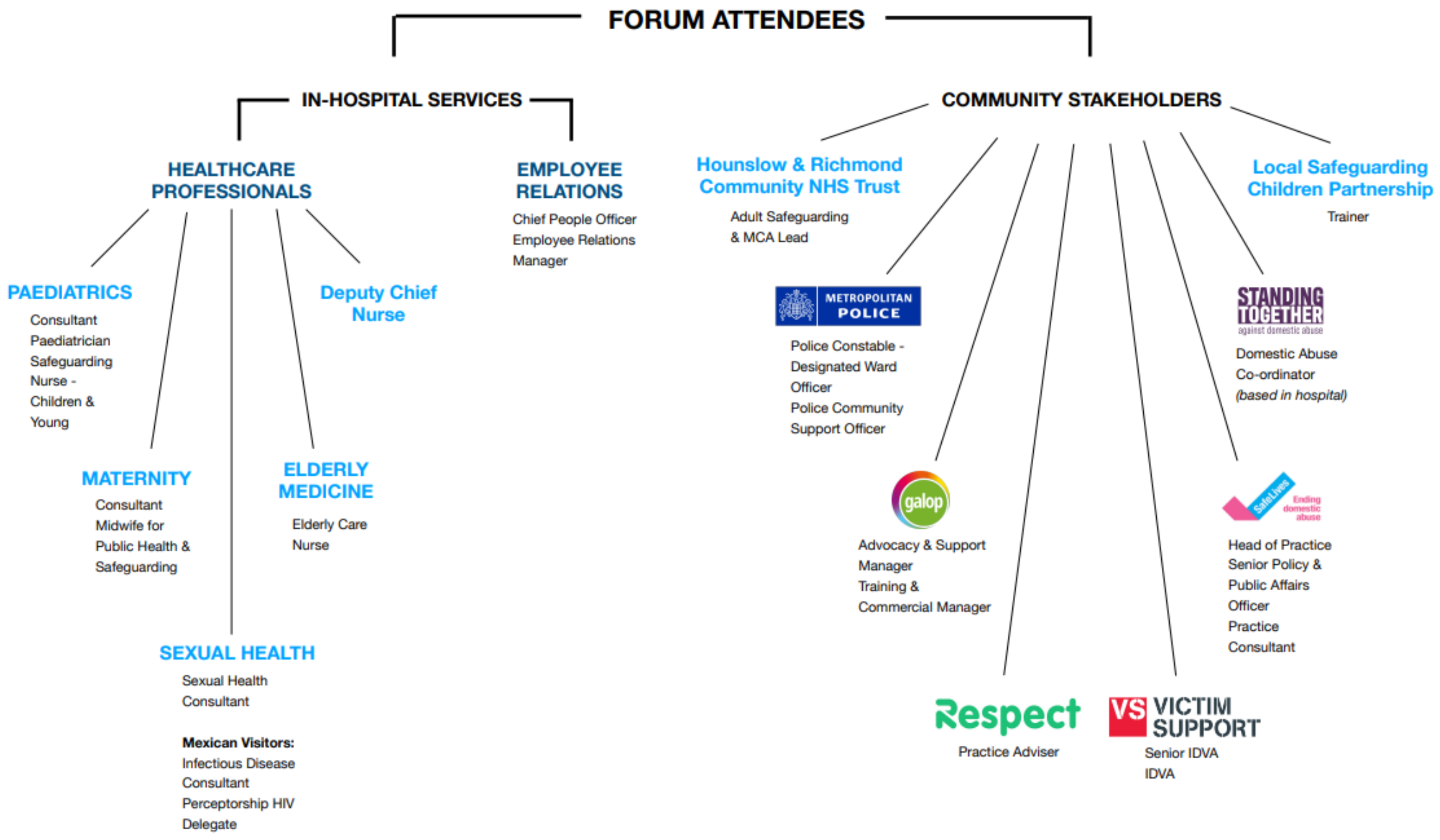

THEMES	FEEDBACK
Positive impact on learning	• **Real-life experience** that **can be manipulated**<br>• Engaging<br>• Supports **retention of learning**<br>• **Remote access** is a key benefit
Bringing lived experience voice	• Bring survivor into the space **without re-traumatising people**<br>• **Co-delivery of scripts**<br>• **Lived experience led session**
Improving identification and communication with DA/SV survivors	• **Reduces unconscious bias**<br>• Used to **identify perpetrators**<br>• Learn how to pick up on behaviours
Improve care of vulnerable patient groups	• Patients with **disability**<br>• Patients with **mental health illness**<br>• **Elderly** patients<br>• **Maternity** cases<br>• **LGBTQ+**<br>• Protecting **children & families** (ie: school settings, family violence, forced marriage)
Training in healthcare	• Nurses<br>• Midwives<br>• Healthcare assistants<br>• Undergraduate education<br>• Paramedics<br>• Staff in GP practices<br>• Social workers<br>• Carehomes<br>• Workers in employee relations

Using the HoloPatient for training on domestic abuse & sexual violence

GigXR

NHS
Chelsea and Westminster Hospital
NHS Foundation Trust

THE FUTURE

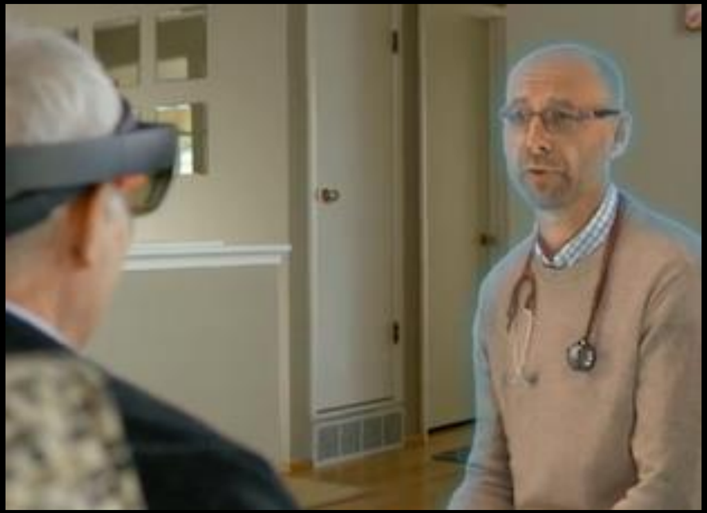

Telehealth using ‘Holo-portation’

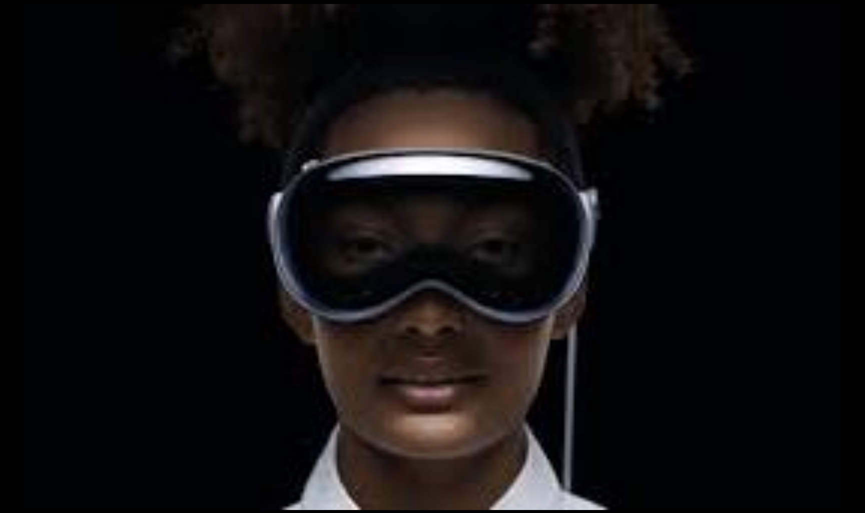

New headsets

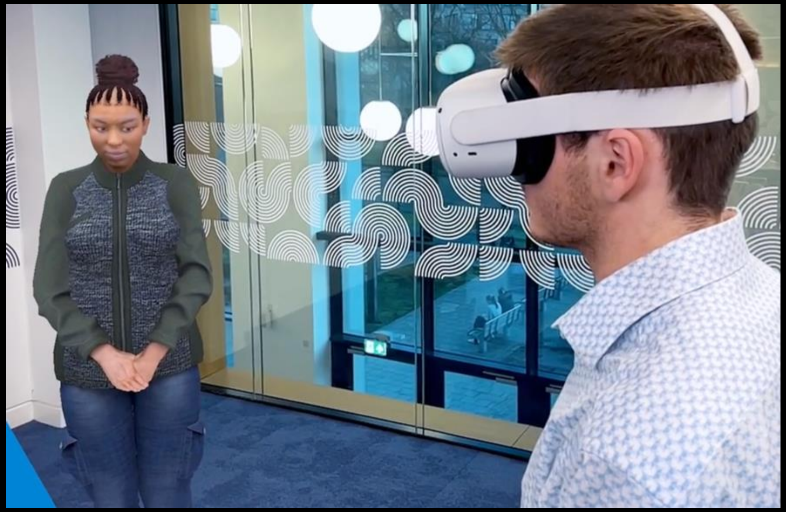

Custom-made avatars

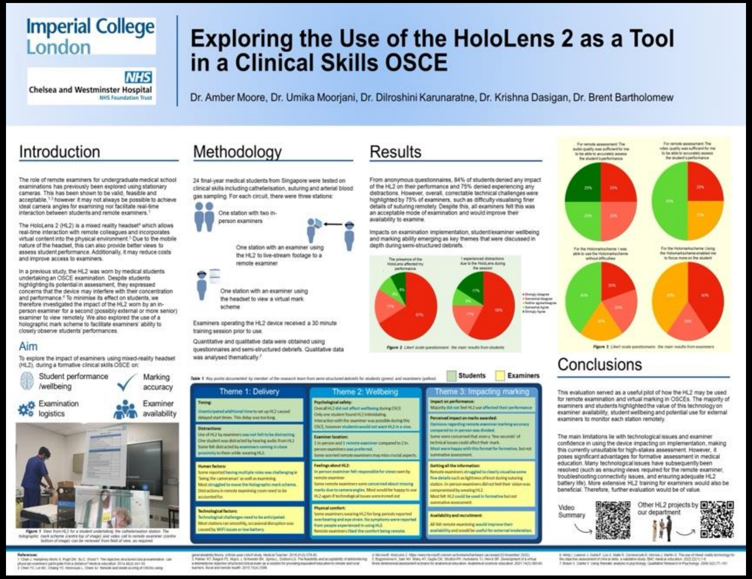

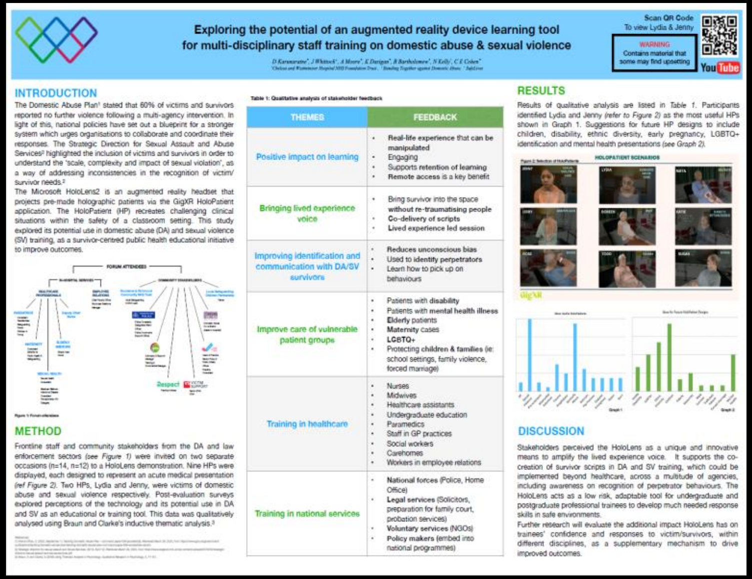

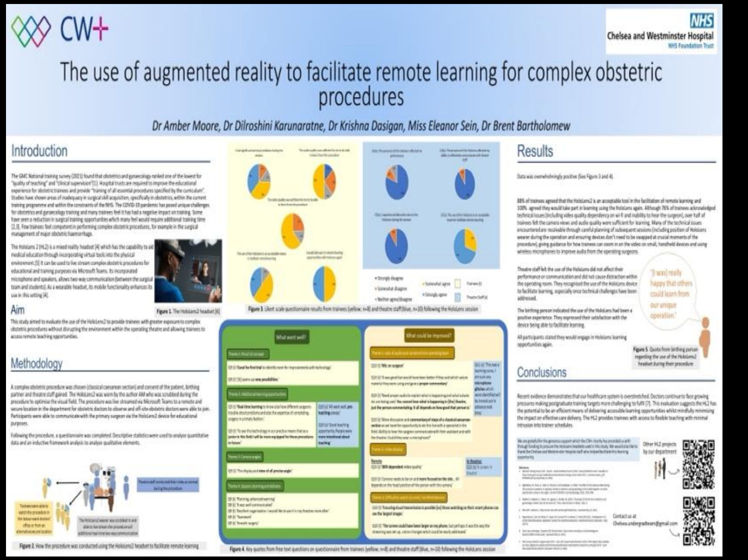

Aug 2022 - Aug 2023

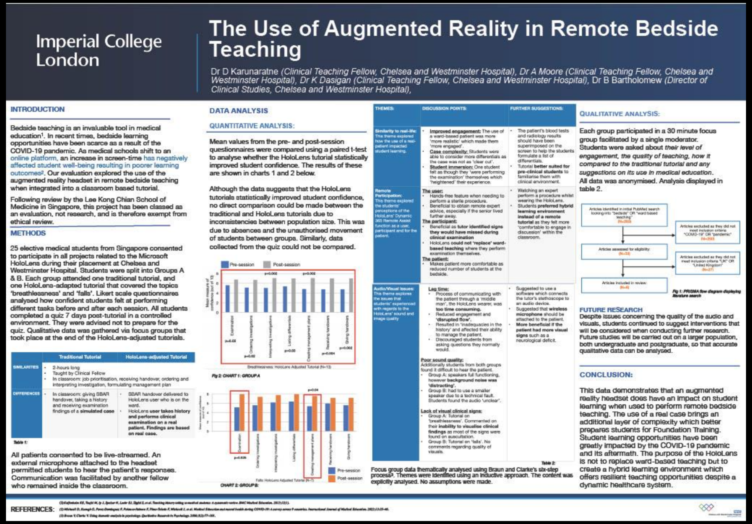

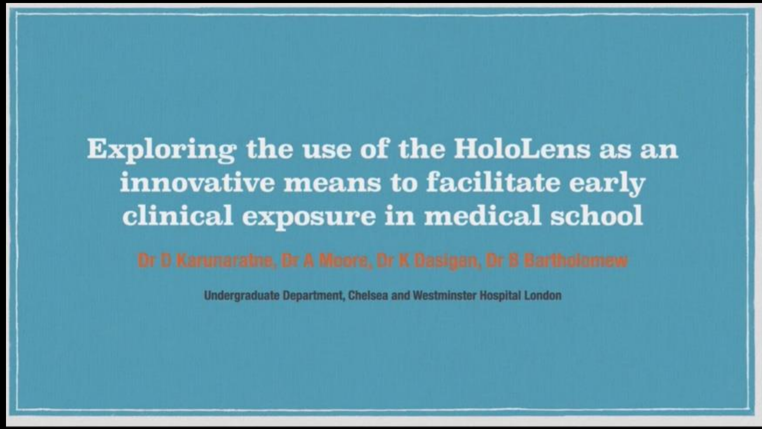

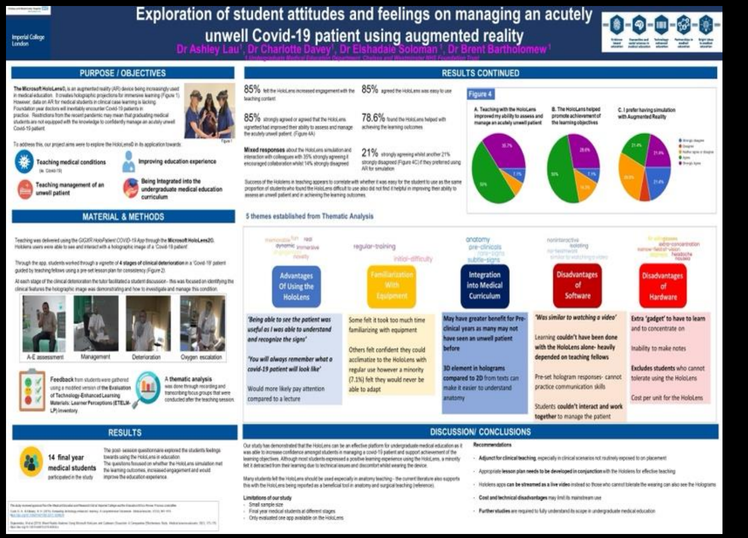

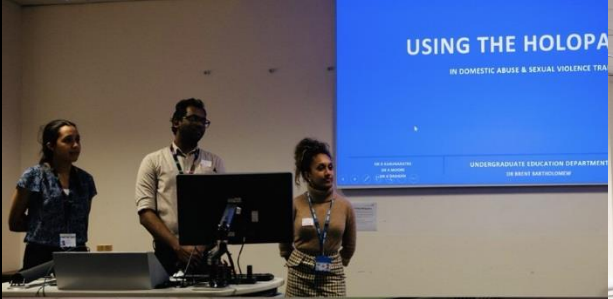

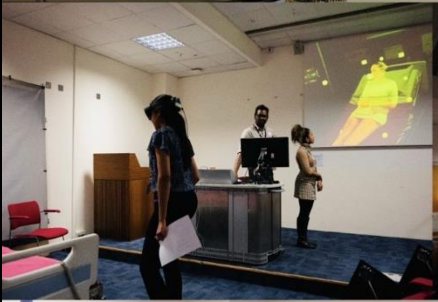

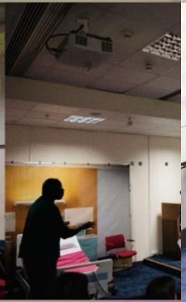

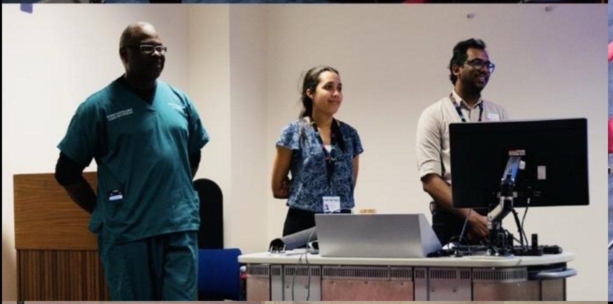

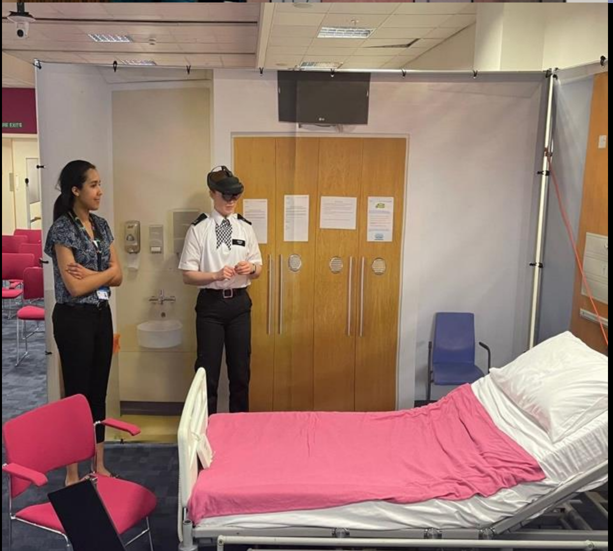

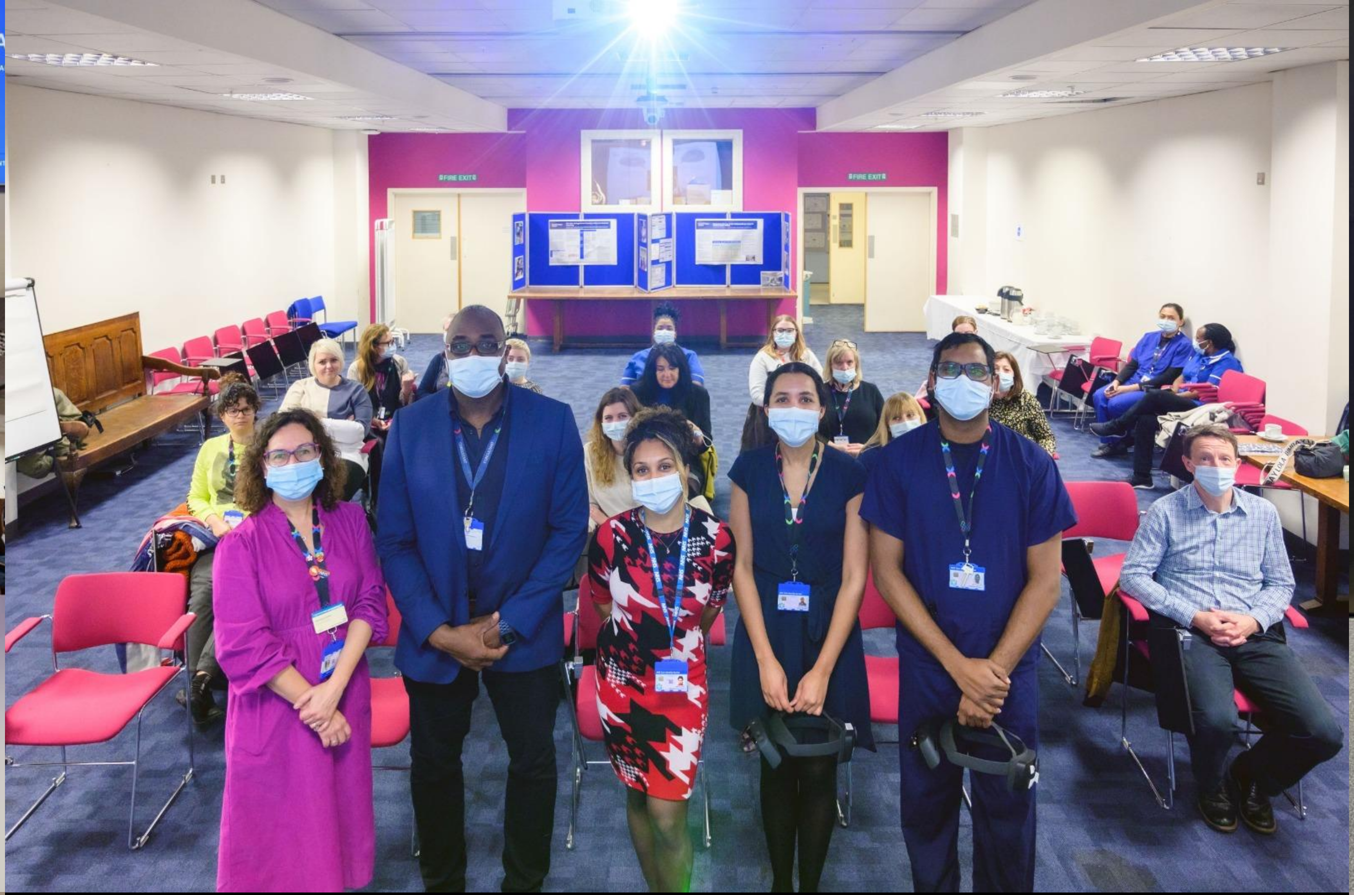

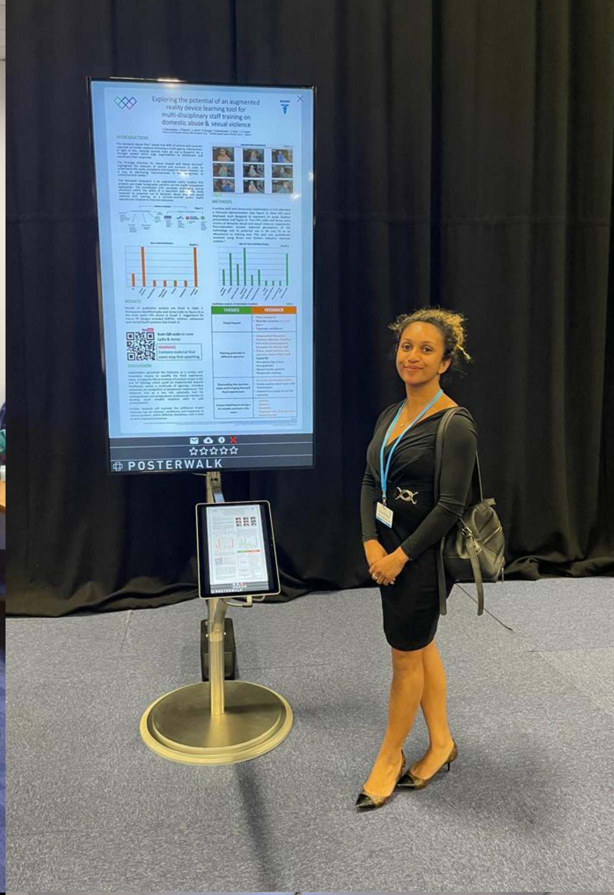

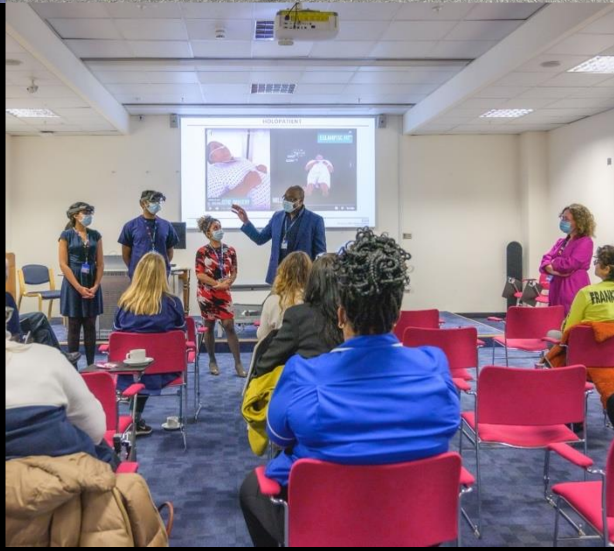

CONTACT US:

Dr Charlotte Cohen & Domestic Abuse Team
Dr Brent Bartholomew & Undergraduate Education Team

chelsea.undergradteam@gmail.com
020 3315 55938

Visit our YouTube page:
Hololens @ Chelwest - YouTube
