# Supplementary material for: Exploring the Use of an Augmented Reality Device Learning Tool for Multidisciplinary Staff Training on Domestic Abuse and Sexual Violence: Postintervention Qualitative Evaluation
Source: JMIR Form Res. 2025 Mar 19;9:e60075. doi: 10.2196/60075 (PMC11941276; doi:10.2196/60075)
Supplement: Multimedia Appendix 4 [file formative-v9-e60075-s004.pdf]

EXPLORING THE USES OF THE HOLOPATIENT IN DOMESTIC ABUSE & SEXUAL VIOLENCE TRAINING

Stakeholder Forum

ORGANISATION: ___________________________

ROLE: _____________________________________

To what extent do you agree with this statement *(circle number to select)*:

1) The HoloLens could be an effective tool used in domestic abuse & sexual violence training?

1 - strongly agree

2 - somewhat agree

3 - neither agree nor disagree

4 - somewhat disagree

5 - strongly disagree

Comments:

2) Current technological limitations with the existing HoloPatients or voice scripts would not prevent me from using this tool in my practice right now.

1 - strongly agree

2 - somewhat agree

3 - neither agree nor disagree

4 - somewhat disagree

5 - strongly disagree

Comments:

3) Which of the holograms that you saw today could be the most useful in domestic abuse and sexual violence training?

Jenny/SV	☐	Lydia/DA	☐	Maya/ Delirium	☐
Jerry/ Anaphylaxis	☐	Doreen/ #NOF	☐	Katie/ DKA	☐
Rose/Sepsis	☐	Todd/ Trauma	☐	Susan/ Burn	☐

4) How could you envisage bringing the lived experience voice into clinical practice using this teaching tool (including to support staff experiencing abuse or violence)? *(eg: re-design HoloPatients, virtual settings, patient scripts etc.)*

5) In health who do you feel this tool is most helpful for? *(eg: medical/nursing students, doctors, nurses, midwives, allied healthcare professionals, GP, admin staff front of house, employee relations etc.)*

6) Outside of health, who do you feel this tool is most helpful for? (eg: *social workers, teachers, police, other etc.)*

7) Are there any common domestic abuse & sexual violence survivor presentations to your area of work, (inclusive of staff), that you would like to see included for the holograms?

8) Where else in the community could you see the HoloLens being used for domestic abuse & sexual violence training?

9) In the community do you feel there would there be value in having a centralised multi-agency HoloLens training hub for professionals to access training?

1 – strongly agree

2 – somewhat agree

3 - neither agree nor disagree

4 – somewhat disagree

5 – strongly disagree

10) If you could design more HoloPatients/HoloStaff/Holograms, what would you like to include on the menu options? List everything you might like to have.

11) Any other comments/suggestions?

This is a Multimedia Appendix to a full manuscript published in the JMIR Formative Research. For full copyright and citation information see http://dx.doi.org/10.2196/jmir.60075.
